# Large-Scale Movements of IF3 and tRNA during Bacterial Translation Initiation

**DOI:** 10.1016/j.cell.2016.08.074

**Published:** 2016-09-22

**Authors:** Tanweer Hussain, Jose L. Llácer, Brian T. Wimberly, Jeffrey S. Kieft, V. Ramakrishnan

**Affiliations:** 1 MRC Laboratory of Molecular Biology, Cambridge CB2 0QH, UK; 2 Department of Biochemistry and Molecular Genetics, University of Colorado Denver School of Medicine, Aurora, CO 80045, USA; 3 RNA BioScience Initiative, University of Colorado Denver School of Medicine, Aurora, CO 80045, USA

**Keywords:** translation, initiation, ribosome, IF1, IF3, IF2, fMet-tRNA, start codon, cryo-EM, structures

## Abstract

In bacterial translational initiation, three initiation factors (IFs 1–3) enable the selection of initiator tRNA and the start codon in the P site of the 30S ribosomal subunit. Here, we report 11 single-particle cryo-electron microscopy (cryoEM) reconstructions of the complex of bacterial 30S subunit with initiator tRNA, mRNA, and IFs 1–3, representing different steps along the initiation pathway. IF1 provides key anchoring points for IF2 and IF3, thereby enhancing their activities. IF2 positions a domain in an extended conformation appropriate for capturing the formylmethionyl moiety charged on tRNA. IF3 and tRNA undergo large conformational changes to facilitate the accommodation of the formylmethionyl-tRNA (fMet-tRNA^fMet^) into the P site for start codon recognition.

## Highlights

• Structures of the 30S ribosomal subunit with initiation factors, tRNA and mRNA
• IF3 helps to position the correct start codon in the P site before binding of tRNA
• Large-scale conformational changes of IF3 and tRNA are observed
• IF3 movements facilitate the accommodation of initiator tRNA in P site

## Graphical Abstract

**Figure:**
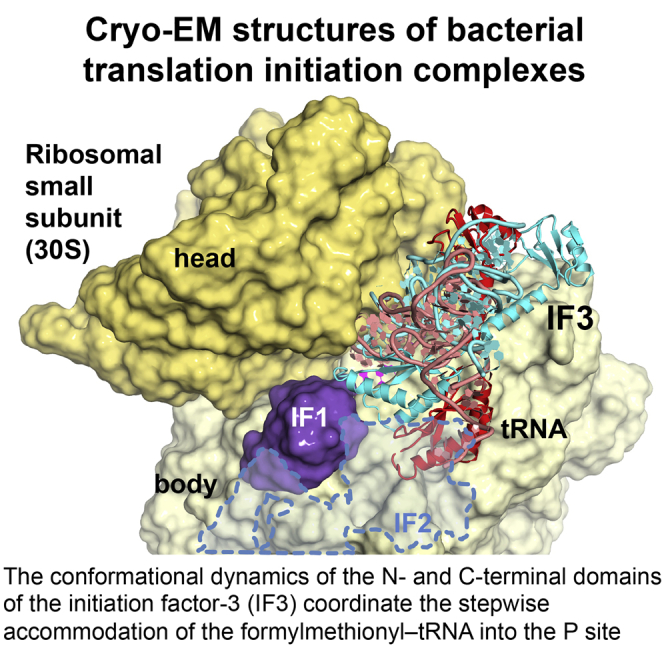

## Teaser

Bacterial ribosomes can start scanning for start codons only after undergoing large scale conformational changes governed by three key initiation factors.

## Introduction

Translational initiation is highly regulated and involves initiation factor (IF)-mediated positioning of an initiator tRNA over an mRNA start codon at the P site of the small ribosomal subunit. In bacteria, translational initiation is controlled by just three IFs, which bind cooperatively to the small ribosomal subunit (30S). IF1 is a small protein that binds in the 30S A site. IF2 is a large GTPase that helps recruit formylmethionyl-tRNA (fMet-tRNA^fMet^), with a C-terminal (C2) domain that specifically recognizes the N-formylmethionine moiety. IF3 is a two-domain protein that plays a particularly important role in the fidelity of selection of tRNA^fMet^ and correct start codon. Although eukaryotic initiation is far more complex, each of these three IFs has a homolog in eukaryotes and in archaea.

In bacteria, the initiation pathway may not necessarily be linear: the order of binding of the factors may be flexible. Binding of many bacterial mRNAs is facilitated by the IF-independent base-pairing of a mRNA Shine-Dalgarno (SD) sequence with the anti-Shine Dalgarno (ASD) sequence at the 3′ end of 16S rRNA. Interaction of a tRNA anticodon with a candidate start codon in the presence of all three IFs defines a 30S pre-initiation complex (PIC). In a poorly understood series of steps, the IFs either directly or indirectly examine the tRNA initiator identity elements and the codon:anticodon interaction. A cognate codon:anticodon interaction is thought to induce a conformational change from the 30S PIC to a 30S initiation complex (30S IC) more favorable for the binding of the 50S subunit to form a 70S initiation complex (70S IC). At some point IF3 dissociates, GTP is hydrolyzed on IF2, followed by dissociation of IF2 and IF1. Initiation ends with an elongation-competent 70S ribosome containing a P-site fMet-tRNA^fMet^ (Milón and Rodnina, 2012, Caban and Gonzalez, 2015).

IF3 discriminates against elongator tRNAs and monitors the codon:anticodon interaction (Hartz et al., 1989, Hartz et al., 1990). IF3 destabilizes tRNA in non-cognate and pseudo-cognate initiation complexes (Petrelli et al., 2001). It is also known to discriminate against initiation of leaderless mRNAs on the 30S subunit (O’Donnell and Janssen, 2002), prevent premature ribosomal subunit association (Grunberg-Manago et al., 1975, Grigoriadou et al., 2007), and render binding of 50S reversible for a short time (MacDougall and Gonzalez, 2015). Several lines of evidence indicate that IF3 adopts multiple functionally important conformations during initiation (Fabbretti et al., 2007, Elvekrog and Gonzalez, 2013, Takahashi et al., 2013). Thus, the discrimination functions of IF3 appear to require large-scale conformational changes.

These complex initiation events in the 30S PIC are not well understood structurally. IF1 is known to enhance the activities of both IF2 and IF3, but the structural basis for this enhancement is not clear from either a high-resolution structure of 30S•IF1 (Carter et al., 2001) or from much lower-resolution cryoEM structures of 30S PICs either without IF3 (Simonetti et al., 2008, Simonetti et al., 2013) or with IF3 (Julián et al., 2011). These lower-resolution reconstructions reveal only a single location and conformation for IF2 and tRNA and weak density interpreted as a single location and conformation for IF3. Thus, structural information is especially limited for IF3, the most interesting and dynamic of the three IFs.

Here, we present 11 structures of *Thermus thermophilus* 30S PIC at resolutions as high as 3.6 Å, representing distinct steps during initiation. IF1 provides key anchoring points for IF2 and IF3, thereby enhancing their activities. IF2 positions its C2 domain in a conformation suitable for binding the formylmethionyl moiety attached to the tRNA^fMet^. We observe large-scale changes in IF3 location and conformation that define distinct roles of IF3 along the initiation pathway. The structures suggest mechanisms for many of the discrimination functions of IF3, including roles in preselecting a good candidate start codon even before tRNA binds, and also in delaying a committed step in ribosomal subunit association.

## Results

### Overview of Structures

**Figure 1:**
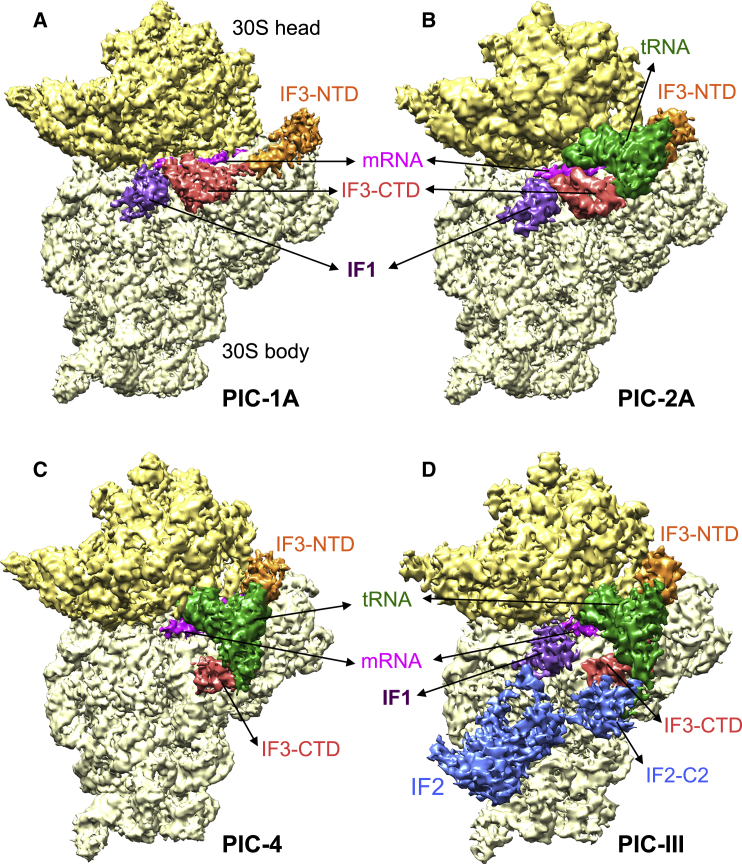
Cryo-EM Maps of 30S PICs (A) PIC-1A containing IF1 (purple), IF3 (NTD, orange; CTD, brick red), and mRNA (magenta). The 30S head is shown in a darker yellow compared to the body. The same color scheme is used in all the figures unless stated otherwise. (B) PIC-2A containing IF1, IF3, mRNA, and fMet-tRNA^fMet^ (green). (C) PIC-4 containing IF3, mRNA, and fMet-tRNA^fMet^. (D) PIC-III containing IF1, IF2 (blue), IF3, mRNA, and fMet-tRNA^fMet^. See also Figures S1, S2, and S3, Tables S1 and S2, and Movie S1.

**Figure S1:**
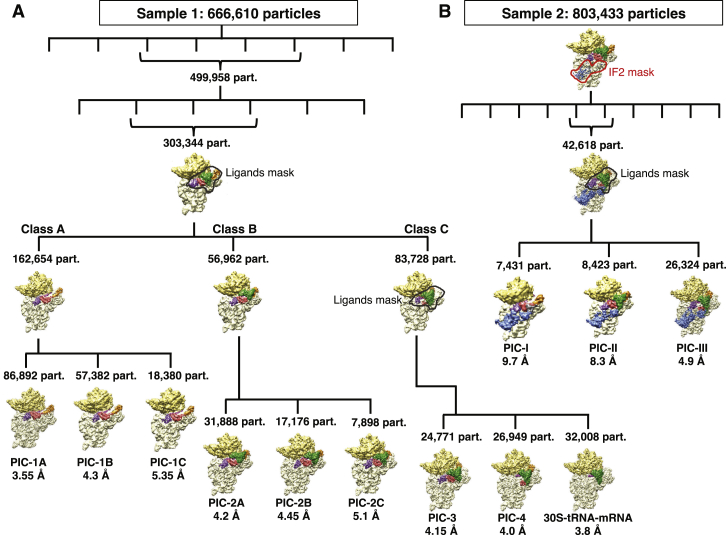
Scheme of 3D Classification of Data, Related to Figure 1 (A) For Sample1 (without IF2) 666,610 particles were selected after 2D classification and an initial 3D refinement was done. The ‘ligands mask’ used for focused 3D classification is shown by an outline. The scheme shows how each PIC was obtained. (See ‘Image processing and structure determination’ in Methods Details for detailed information). (B) For Sample2 (with IF2) 803,433 particles were selected after 2D classification and an initial 3D refinement was done. The ‘IF2 mask’ as well as ‘ligands mask’ used for focused 3D classification is shown by an outline. The scheme shows how PICs- I to III were obtained. (See ‘Image processing and structure determination’ in Methods Details for detailed information).

**Figure S2:**
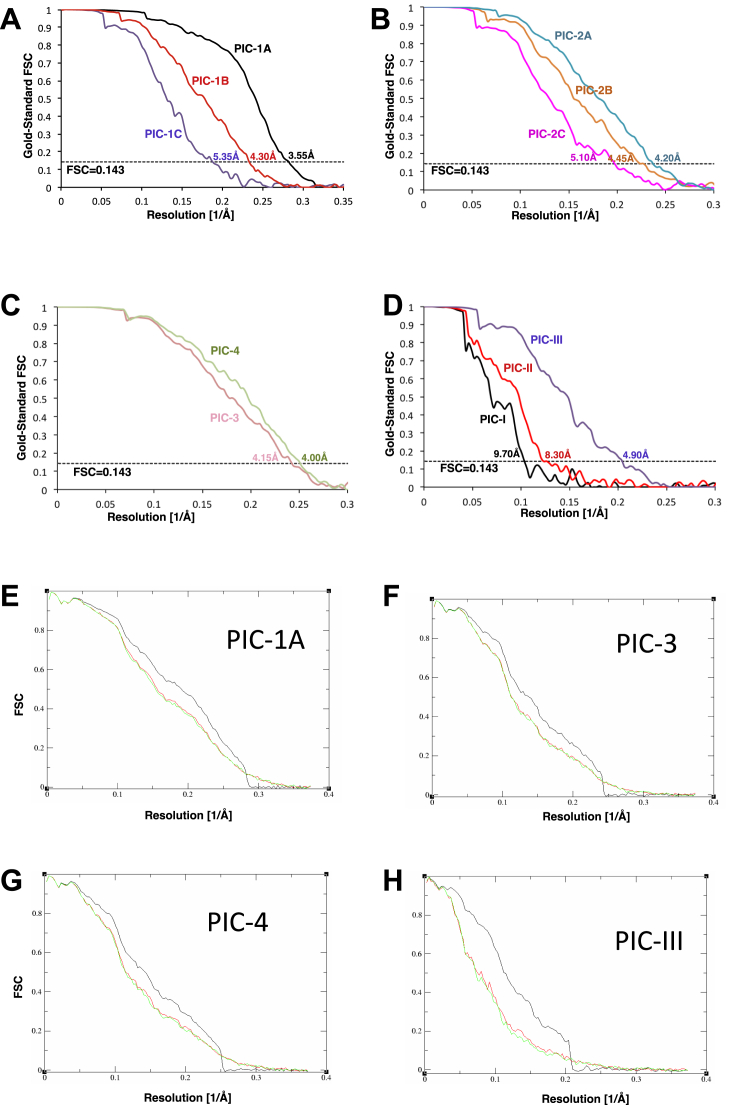
Validation of the PICs, Related to Figure 1 (A) Gold-standard Fourier Shell Correlation (FSC) curves for PICs −1A, 1B and 1C. (B) Gold-standard Fourier Shell Correlation (FSC) curves for PICs −2A, 2B and 2C. (C) Gold-standard Fourier Shell Correlation (FSC) curves for PICs −3 and 4. (D) Gold-standard Fourier Shell Correlation (FSC) curves for PICs -I, II and III. (E) Analysis of overfitting by cross-validation of the PIC-1A model. FSC_work_ curves (red) corresponding to the refined model versus the half-map it was refined against, and FSC_test_ curves (green), i.e., those calculated between the refined atomic model and the other half-map. The black curve shows the FSC curve between a reconstruction from all particles and the model refined against the map. (F) Analysis of overfitting by cross-validation, similar to that in (E), of the PIC-3 model. (G) Analysis of overfitting by cross-validation, similar to that in (E), of the PIC-4 model. (H) Analysis of overfitting by cross-validation, similar to that in (E), of the PIC-III model.

We have determined single-particle cryoEM structures of the 30S PIC from two samples, respectively with and without IF2 (Figures 1, S1, and S2; Table S1). Maps derived from sample 1 (prepared without IF2) are named from PIC 1–4, with the numbering intended to reflect a plausible order of events in the initiation process (see Methods Details for how this was inferred). PICs 1A–1C contain IF1, IF3, and mRNA (but lack tRNA) and differ only in the conformation of the 30S head. PICs 2A–2C contain IF1, IF3, mRNA, and fMet-tRNA^fMet^ and represent states after binding of tRNA but prior to its full accommodation in the P site. PIC-2A shows the initial binding of tRNA at P site in an open conformation of 30S. PIC-2C shows tRNA in a closed conformation of 30S, while PIC-2B represents a likely intermediate step between PIC-2A and 2C. PIC-3 shows a more engaged fMet-tRNA^fMet^, while PIC-4 shows a fully accommodated fMet-tRNA^fMet^ at the P site.

Structures from sample 2 (with IF2) are named PIC-I to III. PIC-I with IF1, IF2, IF3, and mRNA represents a state prior to the binding of tRNA. The positions of IF1 and IF3 are very similar to those observed in the equivalent PICs 1A–1C lacking IF2. This is consistent with the observation of similar intramolecular IF3 smFRET for complexes with and without IF2 (Elvekrog and Gonzalez, 2013). PIC-II contains IF1, IF2, IF3, mRNA, and fMet-tRNA^fMet^ and represents the 30S PIC after binding of tRNA but prior its accommodation in the P site. Here too the positions of IF1 and IF3 are similar to those observed in PICs 2A–2C lacking IF2. PIC-III with IF1, IF2, IF3, mRNA, and fMet-tRNA^fMet^ represents the 30S PIC after accommodation of tRNA in the P site and is very similar to the equivalent IF2-free PIC-4.

**Figure S3:**
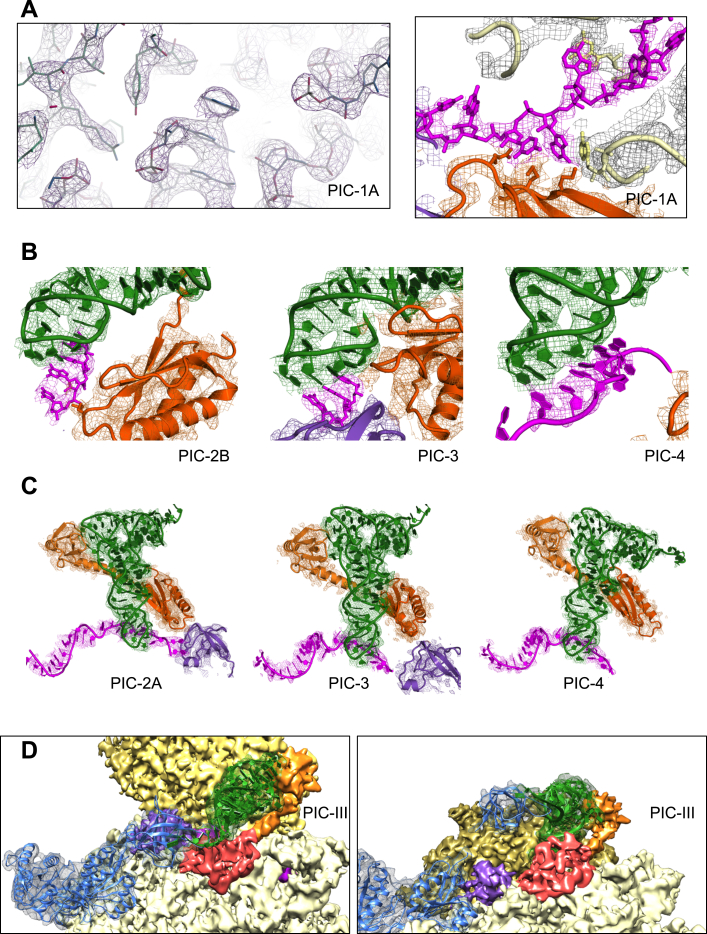
Fitting of Ligands in Density Maps, Related to Figure 1 (A) Left: Representative snapshot showing side chains of ribosomal protein and rRNA fitting in PIC-1A. Right: Fitting of mRNA (magenta), CTD (orange) and rRNA (yellow) at the P site in PIC-1A. (B) Fitting of mRNA (magenta), CTD (orange) and tRNA (green) at the P site in PIC-2B, PIC-3 and PIC-4. (C) Fitting of mRNA (magenta), IF1 (purple), IF3 (orange) and tRNA (green) in PIC-2A, PIC-3 and PIC-4. IF1 is missing in PIC-4. (D) Fitting of IF2 (blue) and tRNA (green) in PIC-III shown in two orientations. IF1 (purple), CTD (brick red), linker/NTD (orange) are also shown.

As observed with other ribosomal complexes, the local resolution and the quality of the density are best in the core of the 30S and in ligands directly attached to it (Table S2). In all structures, distinct density is observed for IFs, tRNA, and mRNA; in the higher resolution structures, density for side chains of proteins are clearly visible (Figure S3; Movie S1). IF1 is observed at the A site in a conformation consistent with the previous crystal structure of 30S•IF1 (Carter et al., 2001). In these structures, we see clear dumbbell-shaped density for IF3, consisting of its N-terminal domain (NTD), C-terminal domain (CTD), and the largely helical linker that separates them (Biou et al., 1995). The CTD binds reversibly near the P site, with conserved residues approaching the codon:anticodon helix, while the NTD binds first to the platform and then to the elbow of fMet-tRNA^fMet^. In each structure, the NTD and CTD occupy different positions near the 30S platform and P site, respectively. Thus, IF3 is observed in distinct conformations in the various 30S PICs, which we propose represent different stages of initiation.

### Rotation of the 30S Head

**Figure 2:**
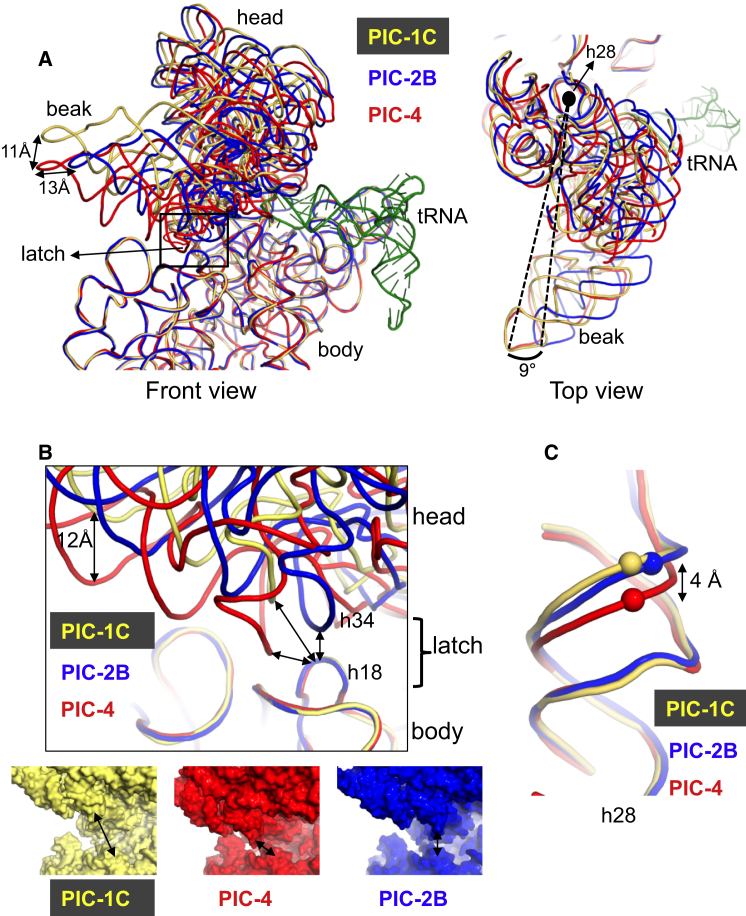
Movement of the 30S Head Widens the mRNA Entry Channel and Opens the Latch (A) Front view of the orientation of the 30S head in PIC-1C (yellow), PIC-2B (blue), and PIC-4 (red), using the 30S body for alignment. The displacement of the beak in the three PICs highlights the difference in the orientation of the head. The P-site tRNA is shown. The top view shows the rotation axis of the head around h28. The magnitudes of the changes are shown using C1030b as the reference point. (B) Front view of superposition of refined models of PIC-1C (yellow), PIC-2B (blue), and PIC-4 (red) indicating elements forming the latch. The movement of 12 Å of A1001 in the 30S head is shown. Bottom panel: surface representation of PICs 1C, 2B, and 4, with the arrows indicating the opening and closing of the mRNA latch. (C) The conformation of h28 in PIC-1C (yellow), PIC-2B (blue), and PIC-4 (red). Equivalent atoms in h28 (C1384) are shown as spheres. In PIC-1C, h28 is not compressed, and the mRNA latch is open. The opposite is seen in PIC-4. In PIC-2B, the mRNA latch is closed by a head swivel rather than compression of h28. See also Table S3 and Movie S2.

A comparison of all 30S PICs presented here shows various positions of the head of 30S with respect to the body, with motions best described as swiveling and/or nodding of the head. An overall rotation of 8–9° of the 30S head along 16S rRNA helix 28 (h28), which forms the “neck” connecting head to the body, is observed among the complexes (Figure 2A; Movie S2). The 30S head is observed with the same canonical swivel seen in 70S ribosomes (Selmer et al., 2006) or eukaryotic initiation complexes (Hussain et al., 2014, Llácer et al., 2015) in most complexes, namely, PICs 1A, 2C, 3, and 4 and PICs I and III (Table S3). In contrast, the 30S head is swiveled in PICs 1B and 2B. The rotation of the head leads to rearrangement of rRNA at the P site as well as movement of the codon:anticodon helix.

Apart from the swiveling of the head, a previously unobserved upward movement of the 30S head is also observed in two structures corresponding to early steps in the pathway (PICs 1C and 2A). This movement opens the A-site mRNA latch formed between h18 and h34 (Schluenzen et al., 2000) (Figure 2B; Movie S2) similar to that observed in an open conformation of eukaryotic initiation complex (Llácer et al., 2015). The h28 is relaxed in the open conformation of 30S, whereas it is compressed in the closed conformation; however, in PIC-2B the 30S head swivel closes the latch, instead of h28 compression (Figure 2C). The mRNA channel is widened from the entry site to at least the P site. The widened cleft as well as opening of the A-site mRNA latch may have implications for loading of mRNA in the channel, as opening the A-site mRNA latch obviates any need for threading mRNA though this latch. We note that a second latch in the E site, formed by S7 and S11, is closed in all of our structures. The body of the 30S subunit does not show any major conformational changes, though subtle conformational changes cannot be ruled out at the current resolutions.

### The Two Domains of IF3 Bind near the P Site and to the Platform

**Figure 3:**
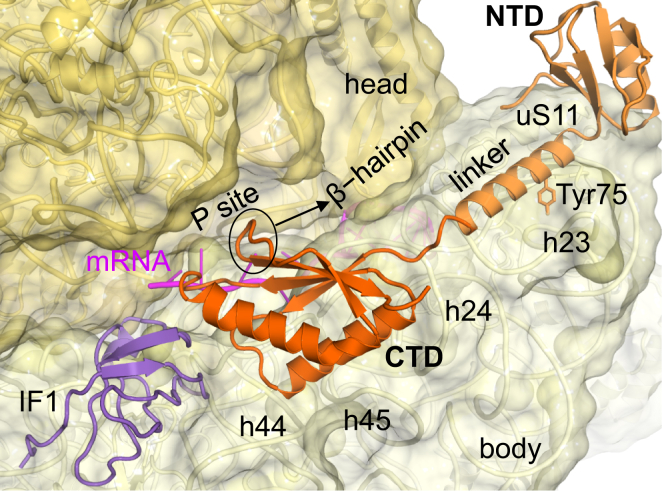
Contacts of IF3 with 30S Ribosomal Subunit The CTD binds next to IF1 at the P site on top of h44 (position 1), and the NTD extends to the platform, while the linker lies on h23 and h24. Tyr75 (*E. coli* numbering) is also shown. See also Figure S4 and Movie S3.

In tRNA-free structures (PICs 1A–1C), the dumbbell-shaped density is seen for nearly all of IF3 (Figure 3). The NTD binds near the 30S platform with a possible interaction with uS11; the bulk of the NTD is positioned away from 16S rRNA. The linker extends toward the P site lying on h23 (687–702) and h24 (787–792) (*Escherichia coli* numbering of 16S rRNA). The CTD density is observed at the top of h44 adjacent to the P site making interactions with both h44 (1494–1497), h24, and with G1517 of h45. The residues of IF3 involved in binding 16S rRNA (63–70, 86–97, 101–105) are mainly hydrophilic and basic. This position of IF3 on 30S agrees with the 16S rRNA cleavage in directed hydroxyl radical probing experiment of 30S⋅IF3 complex (Dallas and Noller, 2001). It is also consistent with protection of rRNA residues in h23 and h24 (G700, U701, G703, G791, and U793) by IF3 in chemical footprints (Muralikrishna and Wickstrom, 1989, Moazed et al., 1995) and other previous mutational studies (de Bellis et al., 1992, Tapprich et al., 1989). Tyr75 (*E. coli* numbering), a NTD/linker residue that when mutated reduces the fidelity of initiation (Maar et al., 2008), is in contact with the platform in these structures.

**Figure S4:**
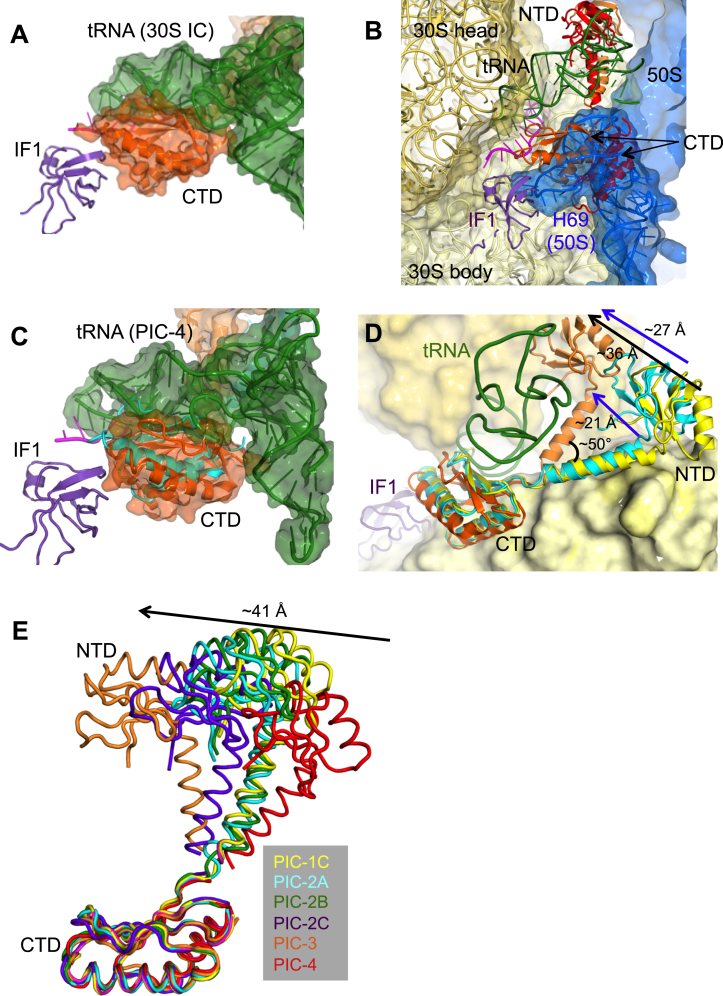
IF3 on 30S, Related to Figures 3 and 4 (A) The CTD in Position1 would clash with fMet-tRNA^fMet^ in a previously reported 30S initiation complex (Simonetti et al., 2008). Only one fMet-tRNA^fMet^ is shown for clarity, as a similar clash is also observed in a subsequent study (Julián et al., 2011). (B) The CTD in Position1 (orange) and Position2 (red) has strong clashes with H69 of large ribosomal subunit (blue) in 70S. The NTD however has no steric hindrance with 50S. (C) The CTD in Position1 (cyan ribbon) and Position1′ (orange surface) would clash with fMet-tRNA^fMet^ (green surface) in PIC-4. (D) Superposition of IF3 in PIC-1C (yellow), PIC-2A (cyan) and PIC-3 (orange) highlighting the movements of the linker (between Asp61) and NTD. The tRNA in PIC-3 is shown. The linker in PIC-3 makes an angle of about 50 degrees with the original position. The NTD moves 28 Å compared to PIC-2A and about 38 Å compared to PIC-1C. (E) Superposition of the CTD of IF3 in PIC-1C (yellow), PIC-2A (cyan), PIC-2B (green), PIC-2C (purple), PIC-3 (orange), PIC-4 (red) highlights the movements of its NTD with respect to it.

The CTD in these complexes is positioned such that it would clash with the tRNA in reported structures of the 30S PIC (Simonetti et al., 2008, Julián et al., 2011; Figure S4A). This P-site location of the CTD (henceforth termed as position 1) features several highly conserved interactions with IF1 as well as with mRNA in the P site. CTD at position 1 also occludes the binding site for H69 of large ribosomal subunit and hence would prevent premature subunit association (Figure S4B).

### Multiple Positions of IF3 in Different tRNA-Bound 30S PICs

**Figure 4:**
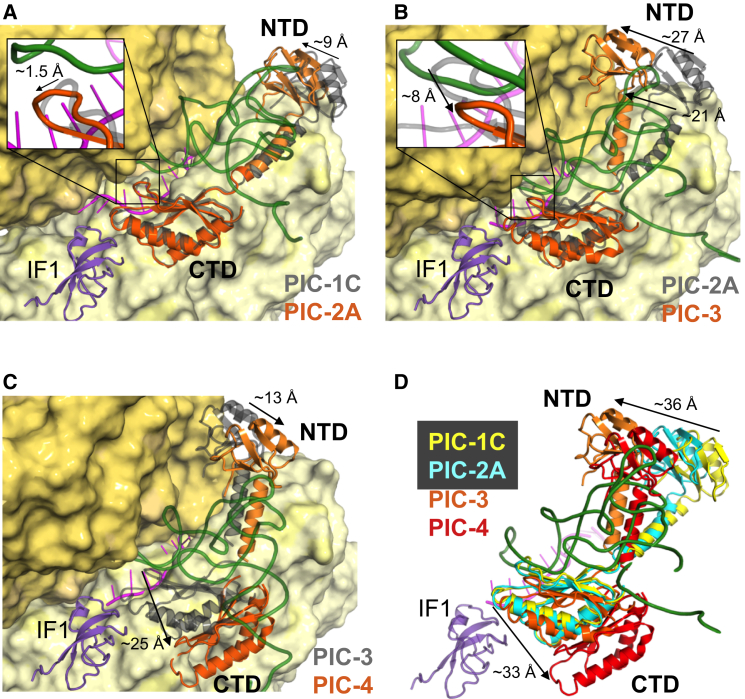
Three Distinct Conformations of IF3 on 30S PICs (A) In PIC-2A, the CTD is in position 1 at the P site, while the NTD moves away (arrow; NTD movement is measured using Arg36 as the reference point) from the platform to bind to the elbow of the fMet-tRNA^fMet^. The linker remains attached to h23 and h24. IF3 from PIC-1C is shown in gray for comparison. Inset shows the movement of the β-hairpin (using Arg159 as the reference) with respect to that in PIC-1C. (B) In PIC-3, the CTD moves slightly to position 1′ at the P site and the NTD moves ∼27 Å while attached to the elbow of the fMet-tRNA^fMet^. The linker is no longer in contact with 30S. IF3 from PIC-2A is shown in gray for comparison. Inset shows the movement of β-hairpin with respect to that in PIC-2A. (C) In PIC-4, the CTD moves ∼25 Å away from the P site to position 2, while the NTD moves while attached to the elbow of the fMet-tRNA^fMet^. The linker is no longer in contact with 30S. IF3 from PIC-3 is shown in gray for comparison. IF1 is not present in PIC-4, but its location in PIC-3 is shown here to highlight the movement of the CTD away from it. (D) Superposition of IF3 in PIC-1C (yellow), PIC-2A (cyan), PIC-3 (orange), PIC-4 (red). The fMet-tRNA^fMet^ and IF1 from PIC-3 are shown to highlight the different conformations of CTD and NTD. The movement of the CTD is measured using Arg125 as the reference point. The arrows show the overall direction of movement of the two domains. See also Figure S4 and Movie S3.

Once tRNA binds, both domains of IF3 appear to sample different positions. Upon binding of fMet-tRNA^fMet^ to an open 30S conformation (PIC-2A), the IF3 NTD moves 9 Å away from the platform to interact with the elbow of tRNA, while the CTD remains in its position at the P site (Figure 4A; Movie S3). Since the open conformation of 30S has a widened P site, the tRNA is not completely engaged in the P site and hence does not have a steric clash with the CTD; only a subtle movement of ∼1.5 Å of the CTD β-hairpin away from the tRNA is seen. The linker too remains almost unchanged.

In PIC-2B and 2C, the CTD remains close to position 1 at the P site with no steric hindrance from the anticodon stem loop (ASL) even though the 30S is in a closed conformation. However, when the fMet-tRNA^fMet^ is accommodated in the P site, as seen in PIC-3 and 4, then bound tRNA would be incompatible with the CTD at position 1 (Figure S4C). In such closed complexes, the CTD is observed in one of two new positions.

In the first tRNA-accommodated CTD location (PIC-3), the CTD rotates around a helix (residues 97–112) to position the β-hairpin away from the P site and fMet-tRNA^fMet^ (Figure 4B; Movie S3). This position of CTD is termed position 1′ (one prime). It appears that the accommodation of the ASL in the P site might push the CTD slightly away from P site, so that it loses the interaction with mRNA. This position is still close to IF1, with a couple of interactions observed between the two proteins, although different from that in position 1. The NTD appears to follow the rotation of the elbow of tRNA as it accommodates into the P site, since it remains in contact with the elbow in both structures. As a result, the NTD has moved further away from the platform (Figure 4B) and about 36 Å from its original position (Figure S4D). This requires the linker connecting to the NTD to rotate by an angle of about 50° from its previous position, with the top of the helix making a displacement of about 21 Å (Figure S4D). In this new position, the linker including Tyr75 has lost most of its contacts with the 30S.

In the structure with a second tRNA-accommodated CTD location (PIC-4), the CTD is observed far away from P site with minimal overlap with either position 1 or 1′. The β-hairpin is now positioned 25 Å away from that of position 1′ (Figure 4C). The CTD now sits lower on h44 (1407–1408 and 1485–1487) and makes more contacts with h45 (1515–1517) and h24 (783–785). In this position, it can no longer contact IF1. This position of CTD is henceforth termed as position 2. The linker is positioned away from the platform with no contacts with 30S. A displacement of about 7 Å is observed from the linker in position 1′. The NTD is observed interacting with the elbow of the fMet-tRNA^fMet^ but is 13 Å away compared to PIC-3. In position 2, the CTD is moved away and hence loses contact with ASL (Figure 4C). The CTD of IF3 observed in very low threshold in a 12-Å map of 30S IC in previous study (Julián et al., 2011) is similar to this position.

Thus, IF3 spans a wide range of conformations in PICs 1–4 as the CTD undergoes remarkable repositioning on 30S (Figures 4D and S4E; Movie S3). Notably, all three locations of the CTD would provide steric hindrance to full association of 50S (Figure S4B). Thus, the CTD may have to completely move away from the 30S to an as yet unobserved position, to allow for the binding of 50S. Throughout its considerable movement, the NTD remains attached to the elbow of the fMet-tRNA^fMet^ in a way that would be compatible with 50S joining, so this may be the last interaction of IF3 in the 70S initiation complex (Figure S4B) prior to its dissociation.

### The SD Sequence and Start Codon in the Presence of IF3

**Figure S5:**
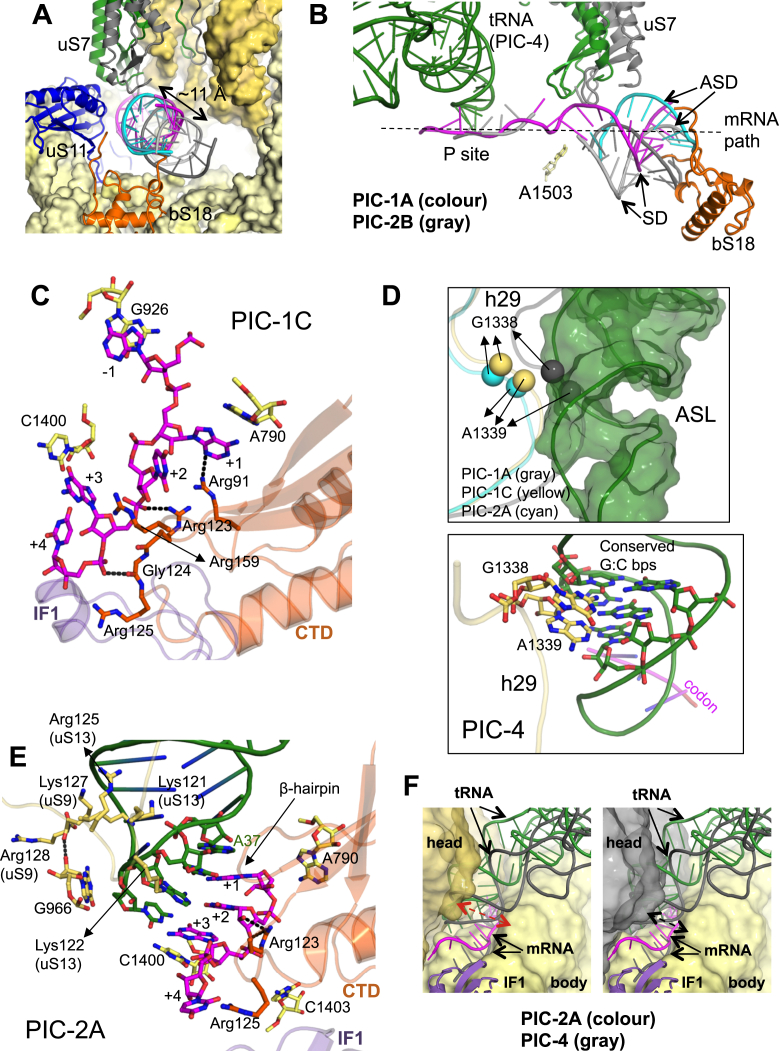
SD/ASD and PICs 1C and 2A, Related to Figure 5 (A) The SD:ASD helix with SD (magenta) and ASD in PIC-1A and PIC-4 (both cyan) and PIC-2B (gray). The SD/ASD in PIC-2B moves ∼11 Å relative to its position in the other PICs. Proteins bS18, uS11 and uS7 are also shown lining the exit channel; The uS7 in PIC-2B (gray) would be too close to the SD/ASD helix in the other PICs. (B) Another view of SD:ASD helices with SD (magenta) and ASD in PIC-1A (cyan) and PIC-2B (gray). A straight mRNA path to the P site is seen for PIC-1A but not for PIC-2B. The uS7 in PIC-2B (gray) would clash with the original position of SD:ASD in PIC-1A. Residue A1503 is also shown away from the mRNA. (C) Interactions of IF3 with the mRNA at the P site in PIC-1C. Arg159 (part of the β-hairpin) is in the vicinity of A+1 and U+2. (D) Top panel: h29 containing G1338 and A1339 (shown as spheres) is positioned optimally in PIC-1C (yellow) similar to that in PIC-2A (cyan) to interact with conserved GCs in ASL of tRNA. However, the h29 (gray) in PIC-1A would clash with ASL. The tRNA from PIC-2A is shown in green surface. Bottom panel: Recognition of conserved GCs in ASL by G1338 and A1339 of h29 in PIC-4. (E) Interactions of IF3 with the mRNA at the P site in PIC-2A. Arg125 of CTD is in the vicinity of the +4 nucleotide of mRNA. The tail of uS9 interacts with G966 and the ASL. The uS13 tail residues also H-bond with the ASL. (F) Comparison of the P site and tRNA and mRNA in PIC-2A (open conformation) and PIC-4 (closed conformation). The 30S, tRNA and mRNA in PIC-2A is shown in color. The tRNA and mRNA in PIC-4 are shown in gray. A widened P site can be seen with the ASL and the codon positioned slightly away from body in the left panel. While in the right panel the 30S head in PIC-4 (in gray) is closer to the body with comparatively narrower P site and the ASL and codon move closer to the body.

A double helix formed by base-pairing between the mRNA SD and ASD is observed in all PICs. The mRNA path and the location of the SD:ASD helix seems to be affected by the presence of IFs (IF1+IF3). In the structures reported here, the SD:ASD helix lies closer to uS11 and uS7 (Figure S5A), and farther from uS2, than in other structures lacking IFs (Jenner et al., 2010). This new and putatively IF-dependent SD:ASD position may impart to the mRNA a roughly straight path from the E site to the P site, which could be important to allow sliding of mRNA for positioning the start codon in the P site. This mRNA path is also more distant from A1503 (Figure S5B), which has been proposed based on some crystal structures to function as a pawl to prevent mRNA slippage just after translocation (Zhou et al., 2013). The SD:ASD helix occupies the same position in all PICs except in PIC-2B where it is slightly displaced from uS7 and uS11 (Figures S5A and S5B).

**Figure 5:**
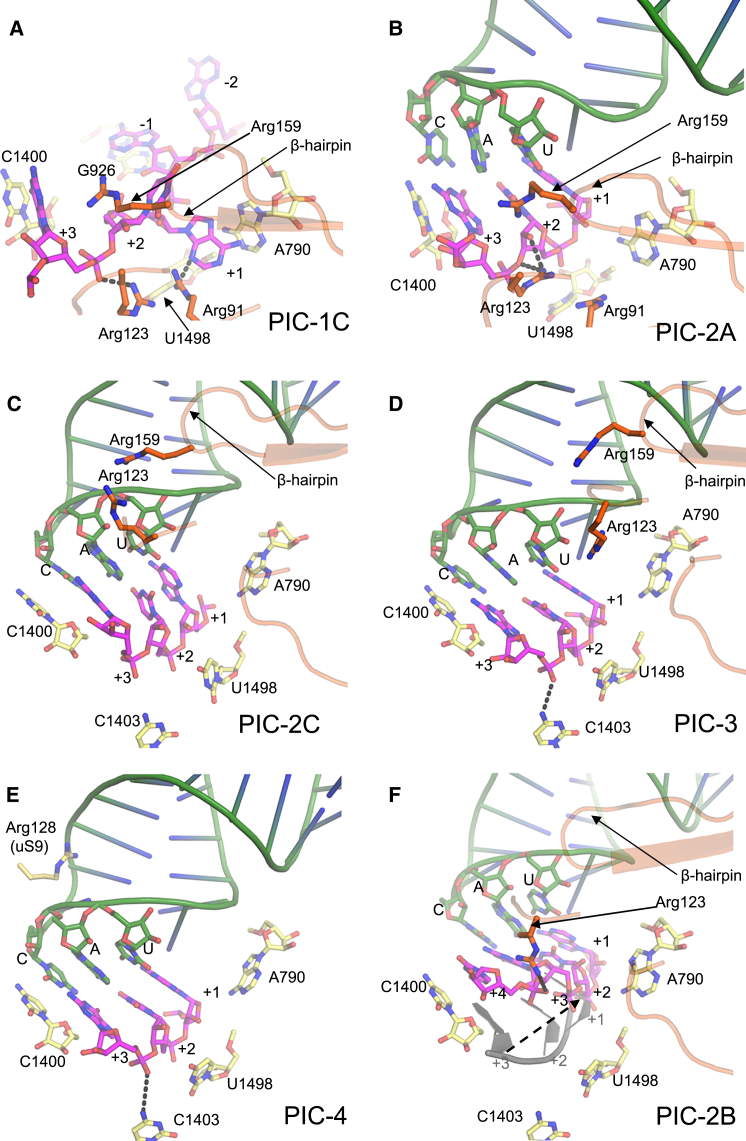
Contacts of IF3 at the P Site (A) In PIC-1C, the A+1 base stacks with A790, while the −1 base stacks with G926. C1400 and U1498 are also shown. Residues of IF3 close to the codon are shown. (B) In the presence of the fMet-tRNA^fMet^ in PIC-2A, the A+1 and U+2 flip to interact with the anticodon. (C) In PIC-2C, the CTD remains in position 1 although no longer close to the codon as seen in PIC-2A. (D) Codon:anticodon interaction in PIC-3 as the CTD is adjusted to position 1′. The Arg123 and Arg159 of IF3 lie close to ASL at the P site. (E) Codon:anticodon interaction in PIC-4 as the CTD is moved away. Arg128 of uS9 interacts with ASL. (F) Similar orientation of the P site in PIC-2B is shown in earlier complexes. The codon:anticodon is moved toward E (dashed arrow) site such that G+3 now occupies the position of A+1. mRNA from PIC-4 is shown in gray. See also Figures S5 and S6 and Movie S4.

In tRNA-free PICs 1A–1C, we observe an unusual mRNA conformation, in which a base at −1 position of mRNA stacks with G926 in the E site, while the next base, the A+1 of the AUG codon, stacks with A790 in the P site (Figure 5A). The base stacking by the −1 base of mRNA is also observed in the case of eukaryotic complexes. The A+1 is flipped toward the 30S body and makes an H-bond with Arg91 of CTD (Figures 5A and S5C). The U+2 of AUG is also flipped and its backbone interacts with Arg123 of CTD. Arg159 of CTD is in the vicinity of A+1 and U+2. The G+3 is not flipped and may interact with C1400. G+3 is in a state ready to base pair with the anticodon, while the +4 nucleotide backbone makes an H-bond with Gly124 of CTD (Figure S5C).

Though the density of the mRNA purine stacked on A790 is most likely A+1, at the present resolution we cannot rule out the possibility that it is G+3 instead. Indeed, this A790 site appears more favorable for binding of G rather than A. It is possible that G+3 binding to this site precedes the A+1 binding we see here, and that IF3 moves the start codon by two nucleotides toward the P site, consistent with the known ability of IF3 to move the mRNA start codon toward the P site (Canonaco et al., 1989, La Teana et al., 1995). Regardless of whether the mRNA purine is A+1 or G+3, these CTD-mRNA interactions suggest a role for IF3 in identifying and stabilizing a good candidate start codon in the P site, even before tRNA binding.

The IF1 and IF3 positions are similar between PICs 1A–1C, which differ only in the conformation of the head. We suggest that PIC-1C is a good candidate for a state just prior to the binding of the fMet-tRNA^fMet^, as it has an open conformation of 30S with a widened P site, and G1338 and A1339 of 16S rRNA are correctly positioned for interactions with the three highly conserved G:C base pairs in the ASL (Figure S5D).

### Conformation of mRNA and tRNA in Different States

There are three structures, PICs 2A–2C, in which fMet-tRNA^fMet^ is not completely accommodated. Among these, PIC-2A has an open conformation of 30S that is most similar to that observed in the preceding state, PIC-1C. We suggest that PIC-2A is a suitable candidate for the first observed state after the binding of the tRNA. The tRNA binds to the 30S head and is placed away from the body in PIC-2A. In the fully accommodated state in PIC-4, the 30S head closes in to narrow the P site and thus the tRNA is positioned very close to body as well as tilted toward it. The intermediate stages of PICs 2C and 3 show a smaller tilt toward the body highlighting the transition of the tRNA from its initial binding position in PIC-2A to the fully accommodated state in PIC-4.

Upon binding of the fMet-tRNA^fMet^, as observed in PIC-2A (Figures 5B and S5E), in an open 30S conformation with a widened P site (Figure S5F), the bases A+1 and U+2 of AUG flip back to base pair with the U36 and A35 of the anticodon, respectively. The CTD Arg123 side chain remains H-bonded to U+2, while the G+3 moves upward to base pair with the anticodon. C1400 in the P site stacks on the G:C base pair, while the residue A37 of tRNA is positioned to stack with the A:U base pair (Figure S5E). Basic residues in the uS9 and uS13 tails are observed interacting with the ASL including the anticodon. The uS9 tail also H-bonds to G966, which, in turn, is observed stacking to the anticodon (Figure S5E). The CTD is observed in position 1 but no direct interaction with ASL is observed. The tRNA in PIC-2A is not accommodated in the widened P site.

**Figure S6:**
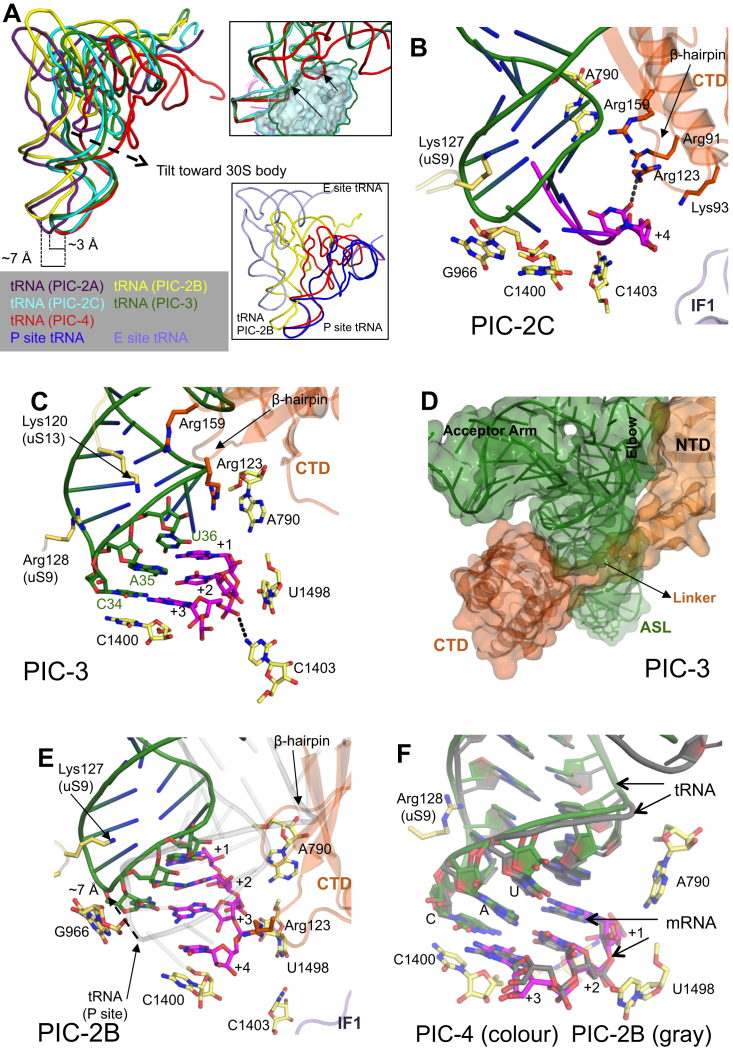
tRNA and IF3 in PICs, Related to Figure 5 (A) The relative movement of the initiator tRNA in the various PICs as deduced by a superposition using the 30S body. The tip of the ASL in PIC-2A (purple) is about 3 Å away from that of PIC-2C (cyan), PIC-3 (green) and PIC-4 (red) while the tip of the ASL in PIC-2B (yellow) is about 7 Å away from the rest. Top Inset: Superposition of PIC-2C (cyan), PIC-3 (green) and PIC-4 (red) using 16S rRNA of the 30S body shows the tilting of the tRNA toward the body. The clashes of the CTD in Position1 (cyan) and Position1′ (green outline) with the tRNAs are also highlighted by arrows. Bottom Inset: tRNA in PIC-4 (red) and in PIC-2B (yellow) are shown with respect to canonical P/P (blue) and E/E (pale blue) tRNAs in the 70S complex, superimposed based on the 30S body. The tRNA in PIC-2B is positioned between the P and E site tRNAs. (B) Interactions of IF3 with the mRNA at the P site in PIC-2C. Arg159 (part of β-hairpin) is in close proximity to ASL. Arg123 of CTD interacts with +4 base of the mRNA. Arg91 and Lys93 of CTD are in vicinity and are also shown. (C) Interactions of IF3 with the mRNA at the P site in PIC-3. Arg159 (part of β-hairpin) is in close proximity to ASL. Arg123 of CTD is also shown. uS9 and uS13 tails interact with ASL. (D) Surface representation of tRNA and IF3 in PIC-3. The CTD and linker is positioned very close to ASL making interactions with it while NTD interacts with elbow of tRNA. (E) tRNA in PIC-2B moves ∼7 Å toward the E site (dashed arrow). The P-site tRNA is shown in transparent gray to highlight the movement. The movement places the tRNA in PIC-2B away from the CTD at the P site. (F) Superposition using the 30S head of PIC-2B (gray) and PIC-4 (color) shows that the codon:anticodon helices superimpose very well at the P site. This shows that the movement of codon:anticodon helix toward the E site in PIC-2B is due to its unique 30S head swivel.

The tRNA in closed conformation of PIC-2C moves closer to the body with the narrowing of the P site compared to PIC-2A. The tip of the ASL in PIC-2C is positioned about 3 Å from that seen in the PIC-2A complex (Figure S6A). Also in PIC-2C, its ASL is tilted toward the body (Figure S6A), and it comes very close to CTD and only a small movement of β-hairpin away from the fMet-tRNA^fMet^ is required to keep CTD in position 1 (Figure 5C). Arg123 of CTD H-bonds with +4 base of mRNA, which may help to keep the mRNA in position, or it might play an indirect role in codon:anticodon discrimination (Figure S6B).

In PIC-3, which presents a state after PIC-2C, fMet-tRNA^fMet^ is almost completely accommodated in the P site (Figures 5D and S6C), and the ASL is tilted about 7° toward the body compared to that in PIC-2C (Figure S6A). The further tilting of the ASL toward the body moves the CTD to adopt position 1′, as the tRNA will clash with the CTD in position 1 (Figure S6A). The CTD and linker are positioned close to the ASL (Figure S6D).

In PIC-4, the last state represented by these PICs, the fMet-tRNA^fMet^ is completely accommodated in the P site in a closed 30S conformation, and the CTD is moved to position 2 (Figure 4C). The ASL in PIC-4 is further tilted 12° toward the body compared to PIC-3 (Figure S6A) and hence would clash with the CTD in either position 1 or position 1′, which explains the need for a distinct position 2 (Figure S4C). The mRNA (codon) is also positioned (3–6 Å) closer to the body compared to PIC-2A (Figure S5F). The codon is held in its place by its interaction with rRNA G926, C1400, C1403, and U1498 in the body (Figure 5E). Based on the open/closed conformation of 30S, the movement of the CTD β-hairpin, and the tilt of the ASL and its accommodation in the P site, the temporal sequence of steps represented by the PICs discussed above is consistent with the following order: PIC-1C, PIC-2A, PIC-2C, PIC-3, and PIC-4.

### A Head Swivel in PIC-2B and Its Implications

Relative to the other structures above, in PIC-2B the head swivels (Figure 2A) to position the ASL ∼7 Å toward the E site (Figures 5F, S6A, and S6E; Movie S4). This movement positions the fMet-tRNA^fMet^ midway between the canonical P- and E-site tRNA as seen in a 70S complex (Figure S6A, inset) (Selmer et al., 2006). Compared to the completely accommodated ASL in PIC-4, this swivel-induced shift is such that the G+3 of the start codon now occupies approximately the position previously occupied by A+1, i.e., a shift of two nucleotides. In addition, the mRNA at the P site is displaced slightly further from the body. Although in PIC-2B, 30S has a closed conformation, h28 is not compressed as in PICs 1A, 2C, 3, and 4 (Figure 2B). The closing of the A-site mRNA latch here is due to swiveling of the head and not the compression of h28. If PIC-2B and PIC-4 are superimposed based on the head, the ASLs are observed in the same position (Figure S6F). This shows that the unusual position of ASL (or codon:anticodon) in PIC-2B is solely due to its tracking with the highly swiveled position of the 30S head. Since tRNA is displaced partly into the E site, it poses no steric hindrance to the observed CTD in position 1.

In line with the above-discussed sequence of steps, one possibility is that PIC-2B may be an intermediate kinetic step between PIC-2A and 2C for destabilizing incorrect tRNAs and correct tRNA^fMet^ bound to poor start codons. Only a swiveling of the PIC-2A head would be required to achieve the PIC-2B state, closing the mRNA latch while keeping h28 relaxed. The highly swiveled head seen in PIC-2B would swivel back, while h28 compresses to achieve a closed conformation of 30S as in PIC-2C. In this scenario, the double head swivel needed to move PIC-2A to PIC-2B to PIC-2C might play an important role in selection of correct tRNA and start codon. Consistent with this possibility, mutations in the neck region increase translation from non-cognate start codon (Qin and Fredrick, 2009).

### Complete 30S PICs with IF2 Are Similar to 30S•IF1•IF3 PICs

**Figure 6:**
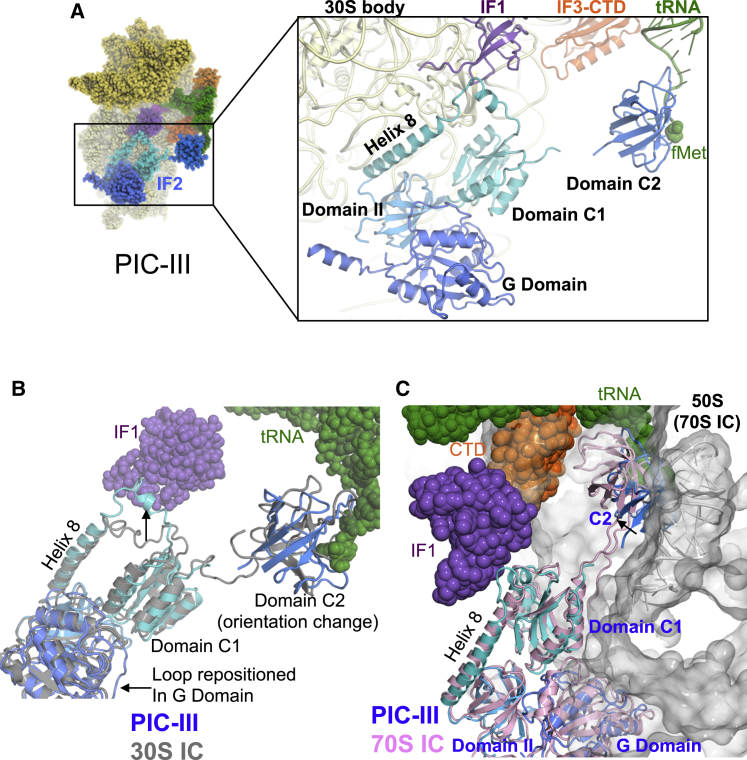
IF2 in 30S PICs (A) Ribbon representation of IF2 in PIC-III highlighting the four domains of IF2 seen in the structures. Each domain is shown is in different shades of blue and labeled. The C-terminal domain C2 interacts with the acceptor arm of the fMet-tRNA^fMet^. The fMet moiety is shown as spheres in its known pocket in C2. (B) Superposition of IF2 fit into previous lower-resolution 30S initiation complex structures (30S IC, gray; Simonetti et al., 2013) with IF2 in PIC-III, showing that the overall position and conformation of IF2 are similar. However, marked differences are seen in the orientation of the C2 domain, the loop connecting helix 8 and domain C1 (that interacts with IF1) and a loop in the G domain. The arrows indicate these differences. (C) Comparison of IF2 in PIC-III (shades of blue) with that in the 70S complex (pink; Sprink et al., 2016) shows a similar position and conformation for most domains of IF2 except C2. The arrow shows that in the 70S there is a movement of C2 toward the 30S body to avoid a clash with 50S (shown in gray). IF1, CTD of IF3 and acceptor arm of fMet-tRNA^fMet^ from PIC-III is also shown. See also Figure S7 and Movie S5.

**Figure S7:**
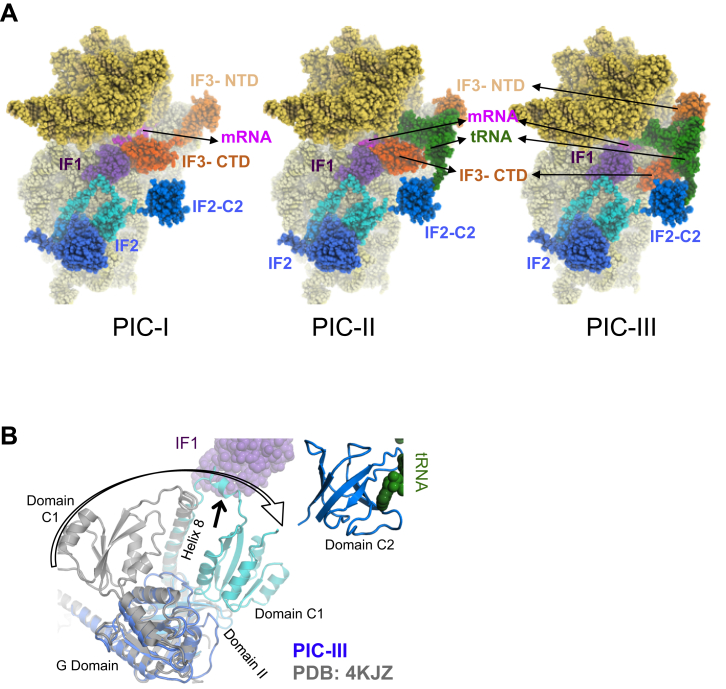
IF2 in 30S PICs, Related to Figure 6 (A) Space filling model of PIC-I, II and III. IF2 is in the same position and conformation in all PICs. (B) Superposition of the crystal structure of *Thermus* IF2 (PDB: 4KJZ; Eiler et al., 2013) on IF2 in PIC-III shows the large movement of domain C1 (curved arrow) upon binding to the 30S. The small arrow shows the loop after helix8 of C1 domain that interacts with IF1 in this new conformation on 30S.

The above structures were derived from sample 1, which omitted IF2 in order to be able to characterize the effects of IF2 binding. We also reconstructed EM maps of 30S PIC from another sample 2 containing all IFs including IF2 (Figure S1). The IF2 conformation is found to be similar in all maps (PICs I–III) irrespective of the presence/absence or position of fMet-tRNA^fMet^ in the P site (Figures 6A and S7A). The C2 domain of IF2 is positioned to bind the CCA of fMet-tRNA^fMet^ even prior to its binding, as seen in PIC-I. In PIC-III, the tRNA^fMet^ is accommodated in the P site, and its CCA end with fMet is observed in the pocket of the C2 (Figure 6A). This conformation of IF2 is different from that in the crystal structure of *Thermus* IF2 (Eiler et al., 2013). In PICs I–III, the rotation of the C1 domain compared to the crystal structure and the conformational change in loop after helix8 of C1 domain allows interaction between IF2 and IF1 (Figure S7B).

The overall conformation of IF2 in this study is similar to that observed in a previous study (Simonetti et al., 2008, Simonetti et al., 2013). However, previously, C2 was modeled in a different orientation in a lower-resolution map than that of PIC-III (Figure 6B). It is likely that a better resolution of PIC-III map may have allowed us to model C2 more accurately, which agrees with the observed density as well as with known studies about the pocket for the acceptor arm of tRNA. It also agrees with the orientation of C2 in a recently reported structure of IF2 in a 70S IC (Sprink et al., 2016), although the position of C2 is different. The C2 observed in PICs I–III here in 30S PICs would clash with the 50S as seen in the superposition of the structures with 70S IC (Sprink et al., 2016) (Figure 6C). Thus, it appears that C2 moves closer to the 30S body upon formation of the 70S IC and the acceptor arm of fMet-tRNA^fMet^ changes from an extended conformation to a more helical one.

The IF2-IF3 interaction is indirect in all three PICs, and contacts are mediated via IF1 or fMet-tRNA^fMet^. In PIC-I, NTD is at the 30S platform, while the CTD is observed in position 1 at the P site (Figure S7A). In PIC-II, NTD now binds to the elbow of the fMet-tRNA^fMet^ away from 30S platform, while the CTD remains in position 1 at the P site (Figure S7A). In PIC-III, NTD remains bound to the elbow of the fMet-tRNA^fMet^ and moves with it, while the CTD moves to position 2 away from P site (Figure S7A). Thus, IF1, IF3, and fMet-tRNA^fMet^ all seem to adopt similar conformations on the 30S in the various states, regardless of the presence or absence of IF2.

## Discussion

**Figure 7:**
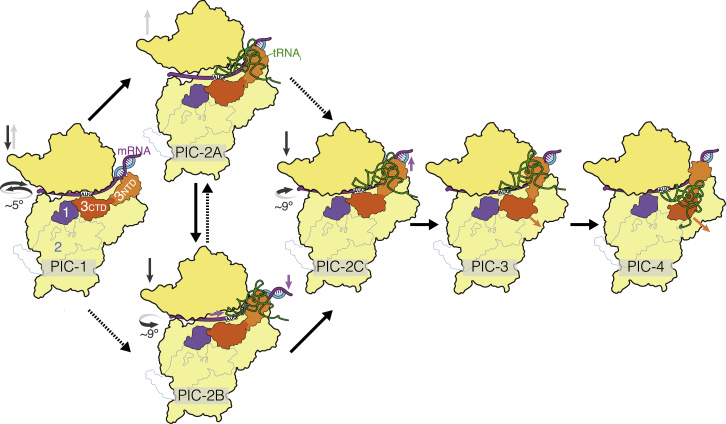
Schematic of Major Conformational Changes during Initiation The various PIC structures are summarized in an order that represents one possible initiation pathway in which mRNA binding precedes tRNA binding. In such an “mRNA-first” pathway, the 30S head may swivel back and forth (shown by curved arrows) and also move up and down (shown by black arrows). The bold arrows show the direction of the pathway. Solid arrows highlight one pathway, while the dashed arrows show possible alternative pathways. The movement of CTD, NTD, and tRNA is shown schematically. See also Movie S5.

All three bacterial IFs cooperate to make translation initiation faster and more accurate. However, a large body of genetic, biochemical, and biophysical data indicate that IF3 plays a particularly important and dynamic role in maintaining the fidelity of initiation. Classic in vitro toeprinting experiments showed that IF3 is necessary and sufficient to discriminate against elongator tRNAs (Hartz et al., 1989) as well as many non-cognate start codons (Hartz et al., 1990). The eight IF2-free structures (PICs 1–4) presented here provide snapshots of different states of the bacterial 30S preinitiation complex with IF3 in different conformational states. Although at lower resolution, the structures of the complete 30S initiation complex (PICs I–III) with all three IFs, fMet-tRNA^fMet^, and mRNA show that the conformations and positions of the tRNA and factors are very similar to those seen in the IF2-free structures, thus validating the conclusions drawn from those higher-resolution structures. Altogether these structures provide many new insights into key stages of translation initiation (Figure 7; Movie S5), and the various IF3 states suggest possible mechanisms for its roles in the translation initiation pathway.

A reasonable “mRNA binds before tRNA” pathway is proposed. In the absence of tRNA, the NTD of IF3 is observed near the 30S platform linked with a long helix to the CTD near the P site at the top of h44. The location of both IF3 domains is consistent with biochemical and mutational studies (Dallas and Noller, 2001, Muralikrishna and Wickstrom, 1989, Moazed et al., 1995, de Bellis et al., 1992, Tapprich et al., 1989). IF3 is observed to adopt at least three different conformations in the structures reported here. It is notable that a single-molecule study also identified dynamic equilibrium between at least three conformations of IF3 during selection of fMet-tRNA^fMet^ and formation of codon:anticodon interaction in 30S initiation complexes (Elvekrog and Gonzalez, 2013). The significance of the multiple conformations of IF3 is discussed below.

In its initial position (position 1), the CTD would clash with tRNA in later, accommodated states; hence, it is clear that the CTD has to move to allow tRNA to be completely engaged in P site. IF1 provides a few highly conserved anchoring points for CTD in this position, in which the tRNA has not yet been “approved.” Thus, IF1 appears to stabilize a more discriminatory state of the CTD, consistent with observations that IF1 and IF3 bind cooperatively.

The interactions between residues in the CTD and the codon in the P site observed before tRNA binding suggest a possible sequence-dependent role for IF3 in directly identifying and stabilizing a good candidate start codon, consistent with the known ability of IF3 to move mRNA (Canonaco et al., 1989, La Teana et al., 1995) as well as data indicating that IF3 in the absence of the other IFs discriminates against some codon:anticodon combinations with Watson-Crick pairing (Hartz et al., 1990). Also, stacking of consecutive mRNA bases with G926 in the E site and with A790 in the P site would also help position the codon for inspection by the anticodon of the tRNA^fMet^.

The codon density in the P site in tRNA-free states PICs 1A–1C is interpreted as AUG with A+1 stacked on A790. With the start codon completely in P site, the initial step of fMet-tRNA^fMet^ binding is likely to be PIC-2A. However, at this resolution we cannot rule out the possibility that the purine stacked on A790 is G+3 rather than A+1. If we interpret this purine as G+3 rather than as A+1, then the start codon would be largely in the E site. In this context, we note that the tRNA in PIC-2B is positioned more in the E site than in the P site, raising the possibility of an alternative sequence of events to the one proposed here. It is also possible that the initial steps in the pathway are not linear, but we propose that all pathways converge onto PIC-2C followed by PIC-3 and PIC-4 (Figure 7). Complementary experiments will be required to identify precisely which pathways are used.

In the three states, PICs 2A–2C, in which the tRNA is not fully accommodated, the CTD remains in position 1 next to the ASL. Irrespective of the relative order of these three states in the pathway, we suggest that these incompletely accommodated states are crucial for the discrimination of the tRNA and codon. Several mutants of IF3 residue Tyr75 confer reduced fidelity but still have full 30S binding affinity (Maar et al., 2008). Because Tyr75 stacks with C701 of h23 only in PICs 1 and 2A–2C and not in later states, it is very likely that important discriminatory steps must precede full accommodation of tRNA. While some of this discrimination may arise from sequence-dependent preselection of good candidate start codons by IF3 before tRNA arrives, we suggest that the highly swiveled state seen in PIC-2B also plays an important role in maintaining fidelity of tRNA and start codon selection. It is possible that this state distorts 30S-tRNA geometry in such a way that the relative importance of interactions common to all tRNAs is reduced.

All three PIC-2 states feature the IF3 CTD in position 1, which would clash with tRNA fully accommodated in the P site. The conformational change in the CTD to allow such accommodation would impose an energetic penalty that could normally be compensated only by a perfect codon:anticodon duplex, thereby promoting initiation accuracy. In PIC-2B, the SD:ASD helix is shifted away from the position observed in other PIC structures here, so that it no longer follows a straight path from the P site that would restrict the movement of the mRNA. It is possible that the 30S PIC may adopt this conformation in order to put further constraint on the codon:anticodon interactions with the CTD at position 1. Thus, only the cognate codon:anticodon interactions that impart enough stability are more likely to remain together, while incorrect or mismatches are more likely to result in dissociation.

The more accommodated tRNA seen in PIC-3 requires the CTD to adopt position 1′. It is interesting to note that CTD Glu151 comes close to the D-loop of tRNA. This and nearby negatively charged residues could lead to a repulsion from the tRNA that leads to positioning the CTD away from the P site following AUG recognition. This is similar to the presence of negatively charged residues in eIF1 positioned closed to the D-loop of initiator tRNA in an accommodated state in yeast (Hussain et al., 2014). It is likely that only when the D-loop/CTD repulsion is overcome by energy from base-pairing of anticodon with the AUG codon that the ASL is stabilized in the P site.

In PIC-3, the two domains of IF3 closely wrap around the tRNA^fMet^ such that the NTD (interacting with the elbow) and CTD (interacting with the D-loop and ASL) come closest to each other in this conformation. In PIC-4, the CTD is moved further away from ASL to position 2, and the two domains of IF3 are furthest apart in this conformation. Although the CTD in positions 1 and 1′ is very close to the ASL, we do not observe a direct monitoring of conserved G:C base pairs of the ASL by IF3. The presence of conserved G:C base pairs in the ASL and its recognition by A-minor interactions by 16S may provide tRNA^fMet^ the additional binding energy to allow it to overcome the steric block as well as electrostatic repulsion of the CTD, thus remaining stably bound where other tRNAs are likely to dissociate.

The linker between the two domains of IF3 adopts a helical structure throughout the initiation pathway. This may be important to coordinate the two separate and distinct movements of the N- and C- domains of IF3. The NTD remains attached to the tRNA elbow, while it rotates from the E to P site and tilts toward the body. The CTD, on the other hand, moves from top of the h44 at the P site to a position away from it and lower on h44. The observed movement of CTD along the initiation pathway may be due to a combination of a steric displacement by the correct ASL and codon at the P site and a pulling action of the NTD as it moves with the elbow of tRNA. With an incorrect tRNA, the CTD would not be displaced by an ASL accommodating into the P site, while the NTD may not bind as tightly to the elbow of a non-initiator tRNA.

In the presence of IF2 in PICs I–III, IF3 adopts the same conformations as observed in complexes without IF2. However, PICs I–III provide insights into the cooperative functioning of the IFs. IF1 shares interactions with both IF2 and IF3 and acts as a bridge between them. The conformations of IF2 in PICS I–III are very similar to one another but different from that of its isolated structure. The conformation of IF2 on the ribosome enables its interaction with IF1, while simultaneously placing the C2 domain in a position to interact with the CCA end of tRNA (even in its absence) as seen in PIC-I. The placement of the C2 domain thus facilitates its binding to the extended CCA of tRNA as seen in PICs II and III. This binding could provide additional specificity for fMet-tRNA^fMet^. However, it has also been suggested that IF2 and initiator tRNA arrive together on the 30S (Tsai et al., 2012), in which case the formylmethionine is pre-checked prior to binding the 30S. A comparison with the recent 70S IC (Sprink et al., 2016) shows that C2 moves toward the 30S body and thus avoid a steric clash with the 50S in the 70S IC. The extended CCA end changes to a more helical conformation in 70S IC.

A comparison of the structures with and without IF2 also shed some light on the dissociation of IF1. In PIC-III, we observe IF1 present in the PIC, while the CTD has moved to position 2. In PIC-4, the equivalent complex without IF2, no density is observed for IF1. Thus, one role of IF2 may be to keep IF1 in place in the later steps of initiation. The absence of IF1 in PIC-4 may be due to h44—an important part of the IF1 binding site—adopting a conformation unfavorable for IF1 once the CTD moves to position 2. This is consistent with previous studies showing changes in h44 in the presence of cognate tRNA and correct start codon (Qin et al., 2012).

The present study sheds light on the multiple steps used to position the correct tRNA and codon during bacterial initiation. It provides insights into the role of each IF, especially of IF3, seen in several previously unobserved states. There are some inherent similarities between this bacterial initiation pathway and eukaryotic initiation. The open and closed conformations of the small subunit and the swiveling of the head play a key role in both. IF1 plays a role similar to eIF1A in eukaryotes in blocking the A site and also providing anchoring points to other factors. This study shows an interesting similarity between IF3-CTD and eIF1, which occupy similar sites in the small subunit and play a crucial role in the fidelity of the process. Both factors have to be displaced from their initial position to allow complete accommodation of tRNA. The IF3-NTD may play a role similar to eIF2α (domain D2) in capturing the elbow of the initiator tRNA. In both cases, the initiator tRNA probably binds in a widened P site and then is subsequently accommodated in a narrowed P site via a series of conformational changes. The binding of the CCA of initiator tRNA is similar in C2 of IF2 and domain IV of the homologous eIF5B. Thus, despite the large differences in the complexity and regulation of initiation in bacteria and eukaryotes, both seem to employ a common core mechanism with the three factors that are common to both kingdoms.

## STAR★Methods

### Key Resources Table

**Table:** 

REAGENT or RESOURCE	SOURCE	IDENTIFIER
**Chemicals, Peptides, and Recombinant Proteins**		

IF1 *Thermus thermophilus*	Carter et al., 2001	N/A
IF2 *Thermus thermophilus*	This paper	N/A
IF3 *Thermus thermophilus*	This paper	N/A
fMet-tRNA *Escherichia coli*	Schmitt et al., 1998	N/A

**Deposited Data**		

EMD-4073	Electron Microscopy Data Bank (EMDB)	http://www.emdatabank.org/
EMD-4074	EMDB	http://www.emdatabank.org/
EMD-4075	EMDB	http://www.emdatabank.org/
EMD-4076	EMDB	http://www.emdatabank.org/
EMD-4077	EMDB	http://www.emdatabank.org/
EMD-4078	EMDB	http://www.emdatabank.org/
EMD-4079	EMDB	http://www.emdatabank.org/
EMD-4080	EMDB	http://www.emdatabank.org/
EMD-4081	EMDB	http://www.emdatabank.org/
EMD-4082	EMDB	http://www.emdatabank.org/
EMD-4083	EMDB	http://www.emdatabank.org/
5LMN	Protein Data Bank (PDB)	http://www.wwpdb.org/
5LMO	PDB	http://www.wwpdb.org/
5LMP	PDB	http://www.wwpdb.org/
5LMQ	PDB	http://www.wwpdb.org/
5LMR	PDB	http://www.wwpdb.org/
5LMS	PDB	http://www.wwpdb.org/
5LMT	PDB	http://www.wwpdb.org/
5LMU	PDB	http://www.wwpdb.org/
5LMV	PDB	http://www.wwpdb.org/

**Experimental Models: Organisms/Strains**		

*Escherichia coli* BL21(DE3)	Invitrogen	Cat#C600003
*Thermus thermophilus*		N/A
*Escherichia coli* K12		N/A

**Recombinant DNA**		

${\text{pBStRNA}}_{Y}^{f\text{Met}}$	Schmitt et al., 1998	N/A
pET13a	Gerchman et al., 1994	N/A
pET30a	Merck Millipore, Novagen	Cat#69909

**Sequence-Based Reagents**		

mRNA sequence: 5′GCUCUUUUAACAAUUUAUCAGGCAAGGAGGUAAAAAUGUUCA-3′	Integrated DNA Technologies	N/A

**Software and Algorithms**		

MotionCorr	Li et al., 2013	http://cryoem.ucsf.edu/software/driftcorr.html
CTFFIND3	Mindell and Grigorieff, 2003	http://grigoriefflab.janelia.org/ctf
Relion-1.4	Scheres, 2012	http://www2.mrc-lmb.cam.ac.uk/relion/index.php/Main_Page
Eman2	Tang et al., 2007	http://blake.bcm.edu/emanwiki/EMAN2
Chimera	Pettersen et al., 2004	https://www.cgl.ucsf.edu/chimera/
Coot v0.8	Emsley et al., 2010	http://www2.mrc-lmb.cam.ac.uk/personal/pemsley/coot/
Refmac v5.8	Brown et al., 2015	https://www2.mrc-lmb.cam.ac.uk/groups/murshudov/content/refmac/refmac.html
Molprobity	Chen et al., 2010	http://molprobity.biochem.duke.edu/
Pymol	DeLano, 2006	http://www.pymol.org

### Contact for Reagents and Resource Sharing

Further information ad requests for reagents may be directed to, and will be fulfilled by the corresponding author V. Ramakrishnan (ramak@mrc-lmb.cam.ac.uk).

### Experimental Model and Subject Details

*Thermus thermophilus* (strain HB8) was grown in ATCC 697 medium consisting of yeast extract, polypeptone peptone, sodium chloride and agar; and adjusted to pH 7.5. The ATCC 697 medium was supplemented with Castenholz salts and the cells were grown under vigorous aeration at 72-75°C in the University of Georgia fermentation facility.

### Methods Details

#### Purification of Ribosomes, mRNA, tRNA, and Initiation Factors

The complete purification of *Thermus thermophilus* 30S ribosomes (McCutcheon et al., 1999) was done at 0-4°C and all buffers contained 6 mM 2-mercaptoethanol, 0.1 mM benzamidine and 0.5 mM phenyl-methyl-sulfonyl fluoride added just before use. The cells were resuspended in buffer (20 mM HEPES, pH 7.5, 10.5 mM Mg(OAc)_2_, 100 mM NH_4_Cl and 0.5 mM EDTA). The cells were passed through the cell disrupter (Emulsiflex) once at 25,000 Psi and the cell lysate was run (centrifugation) in a 45Ti rotor for 30 min at 30,000 rpm. After the spin, the supernatant was carefully collected without disturbing the pellet consisting of cell debris and layered over a sucrose cushion (1.1 M sucrose, 500 mM KCl, 10.5 mM Mg(OAc)_2_, 0.5 mM EDTA and 20 mM HEPES, pH 7.5 in 45Ti tubes. The ribosomes were pelleted overnight at 43,000 rpm for 18.5 hr. The supernatant was removed and the pellet obtained contained ribosomes. The pellet was washed with buffer (1.5 M (NH_4_)_2_SO_4_, 10 mM Mg(OAc)_2_, 400 mM KCl and 20 mM Tris-Cl, pH 7.5) and then resuspended and loaded onto Toyopearl butyl-650S column equilibrated with the same buffer. The 70S was eluted by an inverse gradient of (NH_4_)_2_SO_4_. The fractions containing 70S were collected, pooled and pelleted overnight at 43,000 rpm for 18.5 hr in a sucrose cushion. The 70S pellet was resuspended and loaded on 15%–30% sucrose gradient. This gradient was centrifuged in SW28 rotor at 20,000 rpm for 20.5 hr. After the run, the gradient was fractionated and the 70S containing fractions were collected. Care was taken to remove any 50S containing fractions. The 70S containing fractions were again pelleted overnight at 43,000 rpm for 18.5 hr in a sucrose cushion. The purified 70S was then resuspended in a buffer with only 2.25 mM Mg(OAc)_2_ and loaded on 10%–30% sucrose gradient. This gradient was centrifuged in SW28 rotor at 28,500 rpm for 19 hr to separate out 30S and 50S peaks. After the run, the gradient was fractionated and the 30S containing fractions were collected. Care was taken to remove any 50S containing fractions. The purified 30S was buffer exchanged to a storage buffer (5 mM HEPES, pH 7.5, 50 mM KCl, 10 mM NH_4_Cl and 10 mM Mg(OAc)_2_ and stored as small aliquots in −80°C after flash freezing in liquid nitrogen.

The mRNA oligonucleotide was purchased from Integrated DNA Technologies. The sequence of mRNA was 5′GCUCUUUUAACAAUUUAUCAGGCAAGGAGGUAAAAAUGUUCA-3′ (the codon for fMet is underlined). This sequence is modified from that of Z4C in (Yusupova et al., 2001).

The cells overexpressing fMet-tRNA (Schmitt et al., 1998, Selmer et al., 2006) were grown in LB broth and collected after 16 hr. The pellet was resuspended in lysis buffer (1mM Tris-HCl pH 7.5 and 10mM Mg(OAc)_2_) and routine phenol extraction was performed where the tRNA was ethanol precipitated in the final step. The precipitate was dissolved in Q-sepharose buffer (20 mM tris pH 7.5, 8 mM MgCl_2_, 200mM NaCl and 0.1 mM EDTA) and loaded on Q-sepharose column [pre-equilibrated with the same buffer. The tRNA was eluted with an increasing gradient of NaCl and the fractions containing the tRNA were pooled and dialyzed in the aminoacylation buffer (20 mM tris pH 7.5, 7 mM MgCl_2_ and 150 mM KCl). The tRNA was charged with methionine using methionyl-tRNA synthetase at 37°C for 30 min. The aminoacylation reaction mixture contained 4mM ATP and 200 μM Met. The charged tRNA was formylated with a formyl donor (N^5^-N^10^-methenyl-tetrahydrofolic acid) at a final concentration of 250 μM by addition of 5μM formylase (Methionyl tRNA_f_^Met^ formyl transferase). This reaction was allowed for 30 min at 37°C and then quenched by ethanol precipitation. The formylmethionyl-tRNA pellet was dissolved in buffer containing 10mM NH_4_OAc pH 6.3 and 1.7M (NH_4_)_2_SO_4_ and loaded on TSK Phenyl 5PW column equilibrated with the same buffer. The formylmethionyl-tRNA was eluted with an inverse gradient of (NH_4_)_2_SO_4_. The fractions containing the purified formylmethionyl-tRNA was pooled and dialyzed against storage buffer (10mM NH_4_OAc pH 4.5 and 50mM KCl). Small aliquots were made and flash frozen in liquid nitrogen and stored in −80°C.

IF1 was expressed (Carter et al., 2001) in BL21(DE3) cells grown to A600 = 0.6. The cells were induced with 0.4 mM IPTG and grown for another 3 hr. The cells were collected by centrifugation at 4200 rpm for 20 min and resuspended in lysis buffer (50 mM Tris pH 8.0, 1mM EDTA and protease inhibitor tablet from Roche) and cells were sonicated. The cell lysate was incubated at 65°C to precipitate out the *Escherichia coli* proteins. The precipitate was removed by centrifugation at 20,000 rpm for 30 min. The supernatant, which largely contained IF1 was diluted with ion-exchange loading buffer (50 mM MES pH 6.8, 50 mM KCl and 1 mM DTT) and loaded on an ion-exchange column. The protein was eluted by an increasing KCl gradient. The fractions containing IF1 were collected, pooled and diluted with buffer without KCl and loaded on hydroxylapatite column at pH 6.5. The protein was again eluted by KCl gradient and the fractions containing IF1 were pooled. Finally purified IF1 was buffer exchanged to storage buffer (30 mM HEPES-KOH pH 7.5, 100 mM KCl, 1 mM DTT) and stored as small aliquots in −80°C after flash freezing in liquid nitrogen. The same protocol was used for expression and purification of IF3.

His-tagged IF2 was overexpressed using the T7 expression system (modified pET30a to include a TEV cleavage site) by inducing BL21(DE3) with 1 mM IPTG cells for 4 hr. The cells were collected and resuspended in lysis buffer (0.1 M Tris pH 8, 500 mM KCl, 5 mM BME and Roche protease inhibitor tablet) and cell lysis was carried out by sonication. The sonicated cell lysate was incubated for 30 min at 65°C. Most of the endogenous proteins precipitated and were removed by centrifugation at 10,000 rpm for 25 min. Imidazole was added to supernatant (containing IF2) to 20 mM and pH was adjusted to ∼7.5. It was then loaded onto a Ni-NTA column pre-equilibrated with Ni-NTA loading buffer (50 mM HEPES pH 7.6, 20 mM imidazole, 500 mM KCl and 5 mM BME). The protein was eluted by imidazole gradient and fractions containing IF2 were pooled. The TEV protease was added to remove the N-terminal tag and dialyzed overnight into 50 mM HEPES-KOH pH 7.5, 5 mM BME without KCl. Next, it was loaded onto HiTrap Q column pre-equilibrated with 50 mM HEPES-KOH pH 7.5, 25 mM KCl and 1 mM DTT. IF2 was eluted with a KCl gradient and the fractions containing IF2 were pooled and buffer exchanged to storage buffer (30 mM HEPES-KOH pH 7.5, 30 mM NH4Cl, 5 mM Mg(OAc)_2_, and 1 mM DTT). It was frozen as small aliquots in liquid nitrogen and stored −80°C till further use.

#### Reconstitution of Bacterial Initiation Complexes

A complex at 120 nM (Sample1) was reconstituted by mixing *T. thermophilus* 30S, IF1, IF3, mRNA and fMet-tRNA, in molar ratio of 1:4:4:3:3, in buffer (5 mM HEPES pH 7.5, 10 mM MgAc, 50 mM KCl, 10 mM NH_4_Cl, 6 mM 2-mercaptoethanol). A second complex (Sample2) at 100 nM was prepared by additionally including IF2. In this case, IF2 was preincubated with GDPCP (0.2 mM) and mixed in 30S:IF1:IF2:IF3:tRNA:mRNA molar ratios of 1:3:3:3:3:3, in buffer (10 mM MES pH 6.5, 5 mM MgOAc, 50 mM KCL, 10 mM NH4Cl, 6 mM BME). The samples were used directly to make cryo-EM grids without further purification.

#### Electron Microscopy

3 μl of each complex were applied onto glow-discharged Quantifoil R2/2 cryo-EM grids covered with continuous carbon (of around 50 Å thick) at 4°C and 100% ambient humidity. After a 30 s incubation, the grids were blotted for 3-3.5 s and vitrified in liquid ethane using a Vitrobot Mk3 (FEI).

Automated data acquisitions (EPU software, FEI) were done on Tecnai F30 Polara and Titan Krios microscopes (FEI) at 300 kV for the Sample1 dataset and the Sample2 (IF2-containing dataset), respectively. For the Sample1 dataset, images of 1.1 s/exposure and 17 movie frames were recorded on a Falcon III direct electron detector (FEI) at a calibrated magnification of 104,478 (yielding a pixel size of 1.34 Å). For the Sample2 dataset, images of 1.5 s/exposure and 25 movie frames were recorded on a Falcon II direct electron detector (FEI) at a calibrated magnification of 104,478, resulting in a pixel size of 1.34 Å. For both datasets, dose rates of 27-30 electrons per Å^2^ per second and ranges from 1.5 to 3.0 μm defocus values were used. Micrographs that showed noticeable signs of astigmatism or drift were discarded.

#### Image Processing and Structure Determination

The movie frames were aligned with MOTIONCORR (Li et al., 2013) for whole-image motion correction. Contrast transfer function parameters for the micrographs were estimated using CTFFIND3 (Mindell and Grigorieff, 2003). Particles were picked using RELION (Scheres, 2012). References for template-based particle picking (Scheres, 2015) were obtained from 2D class averages that were calculated from particles picked with EMAN2 (Tang et al., 2007) from a subset of the micrographs. 2D class averaging, 3D classification and refinements were done using RELION-1.4 (Scheres, 2012).

#### Sample 1

For the Sample1 dataset about 4400 images were recorded from five independent data acquisition sessions, and 666,610 particles were selected after two-dimensional classification.

The crystal structure of the 30S of *T. thermophilus* bound to IF1 (PDB: 1HR0) low-pass filtered to 40 Å was used as an initial model for the three-dimensional refinement. After an initial 3D refinement, two consecutive rounds of 3D classification with fine angular sampling and local searches were performed to remove bad particles/empty 30S particles from the data and to get an initial understanding of the conformational heterogeneity of the sample. In the second round of 3D classification, only 3 classes were selected (303,344 particles, 46% of the total) and refined to high resolution.

The preliminary 3D rounds of classification showed 30S in different conformations and tentative positions of IFs and tRNA. Next, we decided to apply a strategy based on the recently reported method of masked classifications with subtraction of the residual signal (Bai et al., 2015), by creating a mask hereafter termed as ‘ligands mask’ based on the densities attributed to the tRNA and IFs in all possible conformations observed in preliminary 3D classification rounds. We used a ‘focused’ 3D classification with this mask to isolate three well-defined types of complexes:

A) Class A showing presence of mRNA, IF1 and IF3 (in Position1) without tRNA [162,654 particles],
B) Class B showing density for mRNA, tRNA, IF1 and IF3 (in Position1) [56,962 particles], and
C) Class C containing mRNA, tRNA, IF1 and IF3 (in Position2 in low occupancy) [83,728 particles].

Class ‘A’ (30S with mRNA, IF1 and IF3 without tRNA) was further classified by standard 3D classification in three classes:

1) 30S in a closed conformation (PIC-1A: 86,892 particles, 3.55 Å)
2) 30S in a closed conformation but with head swiveled (PIC-1B: 57,382 particles, 4.3 Å) and
3) 30S in an open conformation (PIC-1C: 18,380 particles, 5.35 Å).

Class B (30S with mRNA, tRNA, IF1 and in Position1) was also further classified in 3 classes as:

1) 30S in an open conformation (PIC-2A: 31,888 particles, 4.2 Å),
2) 30S in a closed conformation but with head swiveled (PIC-2B: 17,176 particles, 4.45 Å) and
3) 30S in a closed conformation (PIC-2C: 7,898 particles, 5.1 Å).

Class C (30S with mRNA, tRNA, IF1 and in Position2) was further classified into 3 classes using ‘ligand mask’:

1) 30S PIC with mRNA, tRNA, IF1 and IF3 in Position1’ (PIC-3: 24,771 particles; 4.15 Å),
2) 30S PIC with mRNA, tRNA, and IF3 in Position2 (PIC-4: 26,949 particles; 4.0 Å) and
3) 30S PIC with mRNA and tRNA (32,008 particles; 3.8 Å; not discussed in this study)

#### Sample 2

The Sample2 dataset contained about 3200 images and 803,433 particles were selected after 2D classification. An initial 3D refinement was done using the same reference (PDB: 1HR0) as in Sample1 low-pass filtered to 40 Å. Next a masked 3D classification into 10 classes was carried out. The mask around the region on the ribosome where IF2 binds (‘IF2 mask’) based on the low-resolution cryoEM structure of 30S-IF2 (Simonetti et al., 2008); EMD-2448) was used for this 3D classification. Only two classes showed density for IF2 and were subsequently refined to high resolution (42,618 particles, 5.3% of the total, 4.8 Å).

We followed a similar strategy of ‘focused’ 3D classification with ‘ligand mask’ to isolate three well-defined types of complexes:

A) 30S with IF1, IF3 (in Position1) and mRNA without tRNA (PIC-I: 7,431, 9.7 Å),
B) 30S with IF1, IF3 (in Position1), mRNA and tRNA (PIC-II: 8,423 particles, 8.3 Å), and
C) 30S with IF1, IF3 (in Position2), mRNA and tRNA (PIC-III: 26,324 particles 4.9 Å).

Both movie processing (Bai et al., 2013) in RELION-1.4 and particle “polishing” (Scheres, 2014) was performed for all selected particles for 3Drefinement. Resolutions reported here are based on the gold-standard FSC = 0.143 criterion (Scheres and Chen, 2012). All maps were further processed for the modulation transfer function of the detector, and sharpened by applying negative B factors estimated using automated procedures (Rosenthal and Henderson, 2003). Local resolution was estimated using Relion.

#### Naming of Complexes

Maps of PICs obtained from Sample1 (i.e., without IF2) are named from PIC-1 to 4. Maps of PICs obtained from Sample2 (with IF2) are named as PIC-I to III.)

The various PIC structures are named in an order that represents one possible initiation pathway in which mRNA binding precedes tRNA binding. The primary criterion for ordering the structures was to minimize compositional and conformational differences between successive states (see below for details). In such an “mRNA-first” pathway, at least one of the tRNA-free states must occur first, and the final state must be the one with a tRNA fully accommodated in the 30S P site, where “fully accommodated” is defined by comparing with other reported structures.

Things taken into consideration while naming the complexes:

1) 30S conformation: open/closed conformations and swivel of the head
2) Presence or absence of tRNA & its accommodation in the P site
3) IF3-CTD position and conformational changes like position of its β- hairpin relative to ASL
4) Must be more closely related to the previous state or subsequent state than the other complexes
5) Must result in a consistent and reasonable pathway for initiation with minimal conformational excursions

#### Sample 1

PIC-1A contains 30S, IF1, IF3 and mRNA. IF1 is at the A site. IF3- NTD is at the platform while the CTD is at the P site (Position1). The 30S is in the closed conformation with compressed h28. The 30S head is observed in canonical position as in 70S ribosomes (Selmer et al., 2006). The P site is incompatible for the loading of tRNA as it will have a steric hindrance with CTD in Position1.

PIC-1B contains 30S, IF1, IF3 and mRNA. IF1 is at the A site. IF3- NTD is at the platform while the CTD is at the P site (Position1). The 30S is in the closed conformation but h28 is relaxed and the 30S head is swiveled. The P site would be compatible for the loading of tRNA with minor rearrangements. However, the mRNA latch is closed and the P site is narrow.

PIC-1C contains 30S, IF1, IF3 and mRNA. IF1 is at the A site. IF3- NTD is at the platform while the CTD is at the P site (Position1). The 30S is in the open conformation with relaxed h28 and the head is not swiveled. The P site is compatible for the loading of tRNA due to the presence of a widened P site with no obstruction from CTD at Position1.

PIC-2A contains 30S, IF1, IF3, mRNA and fMet-tRNA^fMet^. IF1 is at the A site. IF3- NTD is away from platform and now in contact with elbow of fMet-tRNA^fMet^ while the CTD is at the P site (Position1). The 30S is in the open conformation with a relaxed h28 and the head is not swiveled. The P site is widened. The ASL is tilted away from the 30S body. The CTD is in Position1 with a subtle movement of β- hairpin of IF3 away from ASL.

PIC-2B contains 30S, IF1, IF3, mRNA and fMet-tRNA^fMet^. IF1 is at the A site. IF3- NTD is away from platform and in contact with elbow of fMet-tRNA^fMet^ while the CTD is at the P site (Position1). The 30S is in the closed conformation but h28 is still relaxed and the 30S head is swiveled. The P site is relatively narrowed. The ASL is moved toward the E site. The CTD is in Position1. We have named it as PIC-2B after PIC-2A because a head swivel in PIC-2A would enable the PIC-2B conformation.

PIC-2C contains 30S, IF1, IF3, mRNA and fMet-tRNA^fMet^. IF1 is at the A site. IF3- NTD is away from platform and now is contact with elbow of fMet-tRNA^fMet^ while the CTD is at the P site (Position1). The 30S head is observed in the canonical conformation as in 70S ribosomes (Selmer et al., 2006). The 30S is in closed conformation with h28 compressed. The P site is now narrow. The ASL shows a small tilt toward the body. The CTD is in Position1 with a movement of the β- hairpin of IF3 away from ASL. This movement of the β- hairpin is more than that observed in PIC-2A.

PIC-3 contains 30S, IF1, IF3, mRNA and fMet-tRNA^fMet^. IF1 is at the A site. IF3- NTD is away from platform and in contact with elbow of fMet-tRNA^fMet^ while the CTD is relocated to a slightly different position at the P site (Position1’). The 30S head is observed in canonical position as in 70S ribosomes (Selmer et al., 2006). The 30S is in the closed conformation with h28 compressed. The P site is narrow. The ASL is more accommodated in the P site with a tilt toward the body. The CTD is repositioned to Position1′.

PIC-4 contains 30S, IF3, mRNA and fMet-tRNA^fMet^. IF3-NTD is away from the platform and in contact with elbow of fMet-tRNA^fMet^ while the CTD is relocated away from the P site (Position2). The 30S head is observed in canonical position as in 70S ribosomes (Selmer et al., 2006). The 30S is in the closed conformation with h28 compressed. The P site is narrow. The ASL is most accommodated in this PIC with a maximum tilt toward the body. The CTD is moved to Position2.

#### Sample 2

PIC-I contains 30S, IF1, IF2, IF3 and mRNA. IF1 is at the A site. IF3- NTD is on the platform while the CTD is at the P site (Position1). The 30S head is observed in canonical position as in 70S ribosomes (Selmer et al., 2006).

PIC-II contains 30S, IF1, IF2, IF3, mRNA and fMet-tRNA^fMet^. IF1 is at the A site. IF3- NTD is away from platform and in contact with elbow of fMet-tRNA^fMet^ while the CTD is at the P site (Position1).

PIC-III contains 30S, IF1, IF2, IF3, mRNA and fMet-tRNA^fMet^. IF1 is at the A site. IF3- NTD is in contact with elbow of fMet-tRNA^fMet^ while the CTD is relocated away from P site (Position2). The 30S head is observed in canonical position as in 70S ribosomes (Selmer et al., 2006).

#### Model Building, Validation, and Refinement

The initial model building was done in EM maps with best resolution for the 30S, mRNA or tRNA and for specific IFs. Then this model was used as a reference for model building in EM maps with lower resolution. The head and the body of the atomic model of 30S of *T. thermophilus* (PDB: 1HR0) (Carter et al., 2001) were placed independently into density of each class by rigid-body fitting using Chimera (Pettersen et al., 2004). Next, the crystal structures of IF1 (PDB: 1HR0) (Carter et al., 2001), the N and C-terminal domains of *Geobacillus stearothermophilus* IF3 (PDB: 1TIF and PDB: 1TIG) (Biou et al., 1995), *T. thermophilus* IF2 (PDBs: 3J4J and PDB: 4KJZ) (Simonetti et al., 2013) (Eiler et al., 2013) and tRNA (PDB: 4WZO) (Rozov et al., 2015) were docked into density using Chimera. Then, each chain of the model (including ribosomal proteins, rRNA segments, protein factors and tRNA and mRNA) was rigid-body fitted in Coot (Emsley et al., 2010) and further model building was also done in Coot v0.8.

The availability of crystal structures of N and C-terminal domains of IF3 (PDB: 1TIF and PDB: 1TIG) helped in the model building almost complete IF3 (residue 3 to 170) with the helical linker joining the two domains. Special attention was devoted toward modeling of domain C2 of IF2. Rigid body fitting the NMR structure of C2 of IF2 from *Bacillus stearothermophilus* (PDB: 1D1N) (Meunier et al., 2000) was carried out into the density. Orientation of the C2 domain agrees with previous biochemical data (Guenneugues et al., 2000). It is also in agreement with EM data of its eukaryotic homolog eIF5B (Yamamoto et al., 2014) and with the orientation of C2 resulting from the superimposition on domain C1 of the crystal structure of its archaeal counterpart (PDB: 1G7T) (Meunier et al., 2000). In PIC-III, CCA of tRNA and fMet were taken from (PDB: 1ZO1) (Sprink et al., 2016). Refinement for all but PICs-I and II was carried out in Refmac v5.8 optimized for electron microscopy (Brown et al., 2015), using external restraints generated by ProSMART and LIBG (Brown et al., 2015). Average FSC was monitored during refinement. Final model was validated using MolProbity (Chen et al., 2010). Cross-validation against overfitting was calculated as previously described (Brown et al., 2015, Amunts et al., 2014). Refinement statistics are given in Table S1. All figures were generated using PyMOL (DeLano, 2006) or Chimera.

### Quantification and Statistical Analysis

IF1 concentration was measured by the method of Bradford using commercial reagent from Bio-Rad and bovine serum albumin as a standard. For accurate estimation of the molar concentration of IF2 and IF3, these proteins were quantitated from its optical absorption at 280 nm, using an A_280_^1%^ of 4.10 and 5.77, respectively. The molar absorption value was estimated from the amino acid sequences, using the ProtParam tool of the Expasy database. The 30S ribosomal subunit was measured by its optical absorption at 260 nm.

Resolutions reported here are based on the gold-standard FSC = 0.143 criterion (Scheres and Chen, 2012). All maps were further processed for the modulation transfer function of the detector, and sharpened by applying negative B factors estimated using automated procedures (Rosenthal and Henderson, 2003). Local resolution was also estimated using Relion, using masks of 4 Å around the region of interest, and the same gold-standard FSC = 0.143 criterion. Refinement statistics for each structure, including average FSCs were obtained by Refmac v5.8, optimized for electron microscopy (Brown et al., 2015). Validation statistics were obtained by using MolProbity (Chen et al., 2010).

The 30S head rotation was measured by using h28 (at the neck) as rotation axis and C1030b, at the 30S beak, as the reference point.

### Data and Software Availability

#### Data Resources

Eleven maps have been deposited in the EMDB with accession codes EMDB: 4073, EMDB: 4074, EMDB: 4075, EMDB: 4076, EMDB: 4077, EMDB: 4078, EMDB: 4079, EMDB: 4080, EMDB: 4081, EMDB: 4082 and EMDB: 4083 for PIC-1A, PIC-1B, PIC-1C, PIC-2A, PIC-2B, PIC-2C, PIC-3, PIC-4, PIC-I, PIC-II and PIC-III, respectively. Atomic coordinates have been deposited in the PDB with accession codes PDB: 5LMN, PDB: 5LMO, PDB: 5LMP, PDB: 5LMQ, PDB: 5LMR, PDB: 5LMS, PDB: 5LMT, PDB: 5LMU and PDB: 5LMV for PIC-1A, PIC-1B, PIC-1C, PIC-2A, PIC-2B, PIC-2C, PIC-3, PIC-4 and PIC-III, respectively.

## Author Contributions

T.H., J.L.L., and B.T.W. made the samples, collected and analyzed the data, determined the structures, and wrote a first draft of the manuscript. V.R. supervised the work, and V.R. and J.S.K. helped to write the manuscript.

## Supplemental Information

Document S1. Tables S1–S3

Movie S1. Movie Showing Density for Ligands in the Various EM Maps: PICs 1A, 2A, 4, and III, Related to Figure 1 Densities for all ligands are shown. In PIC-1A, a zoom to the P site is shown along with the density of mRNA and surrounding residues. In PIC-4 we also zoom into the codon:anticodon density. In PIC-III we show a zoomed view of C2 of IF2.

Movie S2. Morphing of PICs 1C, 2B, and 4 to Highlight the Conformational Changes in the 30S Head with Respect to the Body, Related to Figure 2 Both head swivel as well as upward movement of head (which opens up the mRNA latch) is shown. An mRNA (magenta) has been modeled in the mRNA channel. A zoomed view of opening (in PIC-1C) and closing of latch (PIC-2B and 4) is shown. In the neck of the 30S, h28, which relaxes (in PIC-1C) and compresses (PIC-4) during the 30S head movement is also shown.

Movie S3. Movie Highlighting the Conformational Changes in IF3 along the Initiation Pathway, Related to Figures 3 and 4 The 30S head is not shown for the sake of clarity. A zoom to the P site shows the close positioning of IF3 to the mRNA in the absence of tRNA. The β-hairpin closer to tRNA is colored blue to highlight its movement. Surface representations of IF3 and tRNA in a few of the frames show the close positioning of these two ligands in certain steps.

Movie S4. Morphing of PICs 2A, 2B, and 2C to Highlight the Head Swivel, Leading to Movement of tRNA/mRNA toward the E Site, Related to Figure 5 In the zoomed view a canonical E-site tRNA is shown in gray. During the transition from PIC-2A to 2B and subsequently to 2C, the codon in PIC-2A is shown in gray to highlight the movement of codon in PICs-2B and 2C.

Movie S5. Movie Showing the Complete Initiation Pathways, Related to Figures 6 and 7 The movie starts with PIC-I. Thereafter we show the morph of PICs in this order: PIC- 1A, 1B, 1C, 2A, 2B, 2C, 3 and 4. In the end we show PIC-III and how C2 moves when 50S binds taking clue from recent 70S IC structure (Sprink et al., 2016).

## References

[bib1] Amunts A., Brown A., Bai X.C., Llácer J.L., Hussain T., Emsley P., Long F., Murshudov G., Scheres S.H., Ramakrishnan V. (2014). Structure of the yeast mitochondrial large ribosomal subunit. Science.

[bib2] Bai X.C., Fernandez I.S., McMullan G., Scheres S.H. (2013). Ribosome structures to near-atomic resolution from thirty thousand cryo-EM particles. eLife.

[bib3] Bai X.C., Rajendra E., Yang G., Shi Y., Scheres S.H. (2015). Sampling the conformational space of the catalytic subunit of human γ-secretase. eLife.

[bib4] Biou V., Shu F., Ramakrishnan V. (1995). X-ray crystallography shows that translational initiation factor IF3 consists of two compact alpha/beta domains linked by an alpha-helix. EMBO J..

[bib5] Brown A., Long F., Nicholls R.A., Toots J., Emsley P., Murshudov G. (2015). Tools for macromolecular model building and refinement into electron cryo-microscopy reconstructions. Acta Crystallogr. D Biol. Crystallogr..

[bib6] Caban K., Gonzalez R.L. (2015). The emerging role of rectified thermal fluctuations in initiator aa-tRNA- and start codon selection during translation initiation. Biochimie.

[bib7] Canonaco M.A., Gualerzi C.O., Pon C.L. (1989). Alternative occupancy of a dual ribosomal binding site by mRNA affected by translation initiation factors. Eur. J. Biochem..

[bib8] Carter A.P., Clemons W.M., Brodersen D.E., Morgan-Warren R.J., Hartsch T., Wimberly B.T., Ramakrishnan V. (2001). Crystal structure of an initiation factor bound to the 30S ribosomal subunit. Science.

[bib9] Chen V.B., Arendall W.B., Headd J.J., Keedy D.A., Immormino R.M., Kapral G.J., Murray L.W., Richardson J.S., Richardson D.C. (2010). MolProbity: All-atom structure validation for macromolecular crystallography. Acta Crystallogr. D Biol. Crystallogr..

[bib10] Dallas A., Noller H.F. (2001). Interaction of translation initiation factor 3 with the 30S ribosomal subunit. Mol. Cell.

[bib11] de Bellis D., Liveris D., Goss D., Ringquist S., Schwartz I. (1992). Structure-function analysis of *Escherichia coli* translation initiation factor IF3: Tyrosine 107 and lysine 110 are required for ribosome binding. Biochemistry.

[bib12] DeLano, W.L. (2006). The PyMOL Molecular Graphics System. http://www.pymol.org.

[bib13] Eiler D., Lin J., Simonetti A., Klaholz B.P., Steitz T.A. (2013). Initiation factor 2 crystal structure reveals a different domain organization from eukaryotic initiation factor 5B and mechanism among translational GTPases. Proc. Natl. Acad. Sci. USA.

[bib14] Elvekrog M.M., Gonzalez R.L. (2013). Conformational selection of translation initiation factor 3 signals proper substrate selection. Nat. Struct. Mol. Biol..

[bib15] Emsley P., Lohkamp B., Scott W.G., Cowtan K. (2010). Features and development of Coot. Acta Crystallogr. D Biol. Crystallogr..

[bib16] Fabbretti A., Pon C.L., Hennelly S.P., Hill W.E., Lodmell J.S., Gualerzi C.O. (2007). The real-time path of translation factor IF3 onto and off the ribosome. Mol. Cell.

[bib17] Gerchman S.E., Graziano V., Ramakrishnan V. (1994). Expression of chicken linker histones in *E. coli:* Sources of problems and methods for overcoming some of the difficulties. Protein Expr. Purif..

[bib18] Grigoriadou C., Marzi S., Pan D., Gualerzi C.O., Cooperman B.S. (2007). The translational fidelity function of IF3 during transition from the 30 S initiation complex to the 70 S initiation complex. J. Mol. Biol..

[bib19] Grunberg-Manago M., Dessen P., Pantaloni D., Godefroy-Colburn T., Wolfe A.D., Dondon J. (1975). Light-scattering studies showing the effect of initiation factors on the reversible dissociation of *Escherichia coli* ribosomes. J. Mol. Biol..

[bib20] Guenneugues M., Caserta E., Brandi L., Spurio R., Meunier S., Pon C.L., Boelens R., Gualerzi C.O. (2000). Mapping the fMet-tRNA(f)(Met) binding site of initiation factor IF2. EMBO J..

[bib21] Hartz D., McPheeters D.S., Gold L. (1989). Selection of the initiator tRNA by *Escherichia coli* initiation factors. Genes Dev..

[bib22] Hartz D., Binkley J., Hollingsworth T., Gold L. (1990). Domains of initiator tRNA and initiation codon crucial for initiator tRNA selection by *Escherichia coli* IF3. Genes Dev..

[bib23] Hussain T., Llácer J.L., Fernández I.S., Munoz A., Martin-Marcos P., Savva C.G., Lorsch J.R., Hinnebusch A.G., Ramakrishnan V. (2014). Structural changes enable start codon recognition by the eukaryotic translation initiation complex. Cell.

[bib24] Jenner L.B., Demeshkina N., Yusupova G., Yusupov M. (2010). Structural aspects of messenger RNA reading frame maintenance by the ribosome. Nat. Struct. Mol. Biol..

[bib25] Julián P., Milon P., Agirrezabala X., Lasso G., Gil D., Rodnina M.V., Valle M. (2011). The Cryo-EM structure of a complete 30S translation initiation complex from *Escherichia coli*. PLoS Biol..

[bib26] La Teana A., Gualerzi C.O., Brimacombe R. (1995). From stand-by to decoding site. Adjustment of the mRNA on the 30S ribosomal subunit under the influence of the initiation factors. RNA.

[bib27] Li X., Mooney P., Zheng S., Booth C.R., Braunfeld M.B., Gubbens S., Agard D.A., Cheng Y. (2013). Electron counting and beam-induced motion correction enable near-atomic-resolution single-particle cryo-EM. Nat. Methods.

[bib28] Llácer J.L., Hussain T., Marler L., Aitken C.E., Thakur A., Lorsch J.R., Hinnebusch A.G., Ramakrishnan V. (2015). Conformational Differences between Open and Closed States of the Eukaryotic Translation Initiation Complex. Mol. Cell.

[bib29] Maar D., Liveris D., Sussman J.K., Ringquist S., Moll I., Heredia N., Kil A., Bläsi U., Schwartz I., Simons R.W. (2008). A single mutation in the IF3 N-terminal domain perturbs the fidelity of translation initiation at three levels. J. Mol. Biol..

[bib30] MacDougall D.D., Gonzalez R.L. (2015). Translation initiation factor 3 regulates switching between different modes of ribosomal subunit joining. J. Mol. Biol..

[bib31] McCutcheon J.P., Agrawal R.K., Philips S.M., Grassucci R.A., Gerchman S.E., Clemons W.M., Ramakrishnan V., Frank J. (1999). Location of translational initiation factor IF3 on the small ribosomal subunit. Proc. Natl. Acad. Sci. USA.

[bib32] Meunier S., Spurio R., Czisch M., Wechselberger R., Guenneugues M., Gualerzi C.O., Boelens R. (2000). Structure of the fMet-tRNA(fMet)-binding domain of *B. stearothermophilus* initiation factor IF2. EMBO J..

[bib33] Milón P., Rodnina M.V. (2012). Kinetic control of translation initiation in bacteria. Crit. Rev. Biochem. Mol. Biol..

[bib34] Mindell J.A., Grigorieff N. (2003). Accurate determination of local defocus and specimen tilt in electron microscopy. J. Struct. Biol..

[bib35] Moazed D., Samaha R.R., Gualerzi C., Noller H.F. (1995). Specific protection of 16 S rRNA by translational initiation factors. J. Mol. Biol..

[bib36] Muralikrishna P., Wickstrom E. (1989). *Escherichia coli* initiation factor 3 protein binding to 30S ribosomal subunits alters the accessibility of nucleotides within the conserved central region of 16S rRNA. Biochemistry.

[bib37] O’Donnell S.M., Janssen G.R. (2002). Leaderless mRNAs bind 70S ribosomes more strongly than 30S ribosomal subunits in *Escherichia coli*. J. Bacteriol..

[bib38] Petrelli D., LaTeana A., Garofalo C., Spurio R., Pon C.L., Gualerzi C.O. (2001). Translation initiation factor IF3: Two domains, five functions, one mechanism?. EMBO J..

[bib39] Pettersen E.F., Goddard T.D., Huang C.C., Couch G.S., Greenblatt D.M., Meng E.C., Ferrin T.E. (2004). UCSF Chimera—a visualization system for exploratory research and analysis. J. Comput. Chem..

[bib40] Qin D., Fredrick K. (2009). Control of translation initiation involves a factor-induced rearrangement of helix 44 of 16S ribosomal RNA. Mol. Microbiol..

[bib41] Qin D., Liu Q., Devaraj A., Fredrick K. (2012). Role of helix 44 of 16S rRNA in the fidelity of translation initiation. RNA.

[bib42] Rosenthal P.B., Henderson R. (2003). Optimal determination of particle orientation, absolute hand, and contrast loss in single-particle electron cryomicroscopy. J. Mol. Biol..

[bib43] Rozov A., Demeshkina N., Westhof E., Yusupov M., Yusupova G. (2015). Structural insights into the translational infidelity mechanism. Nat. Commun..

[bib44] Scheres S.H. (2012). RELION: Implementation of a Bayesian approach to cryo-EM structure determination. J. Struct. Biol..

[bib45] Scheres S.H. (2014). Beam-induced motion correction for sub-megadalton cryo-EM particles. eLife.

[bib46] Scheres S.H. (2015). Semi-automated selection of cryo-EM particles in RELION-1.3. J. Struct. Biol..

[bib47] Scheres S.H., Chen S. (2012). Prevention of overfitting in cryo-EM structure determination. Nat. Methods.

[bib48] Schluenzen F., Tocilj A., Zarivach R., Harms J., Gluehmann M., Janell D., Bashan A., Bartels H., Agmon I., Franceschi F., Yonath A. (2000). Structure of functionally activated small ribosomal subunit at 3.3 angstroms resolution. Cell.

[bib49] Schmitt E., Panvert M., Blanquet S., Mechulam Y. (1998). Crystal structure of methionyl-tRNAfMet transformylase complexed with the initiator formyl-methionyl-tRNAfMet. EMBO J..

[bib50] Selmer M., Dunham C.M., Murphy F.V.T., Weixlbaumer A., Petry S., Kelley A.C., Weir J.R., Ramakrishnan V. (2006). Structure of the 70S ribosome complexed with mRNA and tRNA. Science.

[bib51] Simonetti A., Marzi S., Myasnikov A.G., Fabbretti A., Yusupov M., Gualerzi C.O., Klaholz B.P. (2008). Structure of the 30S translation initiation complex. Nature.

[bib52] Simonetti A., Marzi S., Billas I.M., Tsai A., Fabbretti A., Myasnikov A.G., Roblin P., Vaiana A.C., Hazemann I., Eiler D. (2013). Involvement of protein IF2 N domain in ribosomal subunit joining revealed from architecture and function of the full-length initiation factor. Proc. Natl. Acad. Sci. USA.

[bib53] Sprink T., Ramrath D.J., Yamamoto H., Yamamoto K., Loerke J., Ismer J., Hildebrand P.W., Scheerer P., Bürger J., Mielke T., Spahn C.M. (2016). Structures of ribosome-bound initiation factor 2 reveal the mechanism of subunit association. Sci. Adv..

[bib54] Takahashi S., Isobe H., Ueda T., Okahata Y. (2013). Direct monitoring of initiation factor dynamics through formation of 30S and 70S translation-initiation complexes on a quartz crystal microbalance. Chemistry.

[bib55] Tang G., Peng L., Baldwin P.R., Mann D.S., Jiang W., Rees I., Ludtke S.J. (2007). EMAN2: An extensible image processing suite for electron microscopy. J. Struct. Biol..

[bib56] Tapprich W.E., Goss D.J., Dahlberg A.E. (1989). Mutation at position 791 in *Escherichia coli* 16S ribosomal RNA affects processes involved in the initiation of protein synthesis. Proc. Natl. Acad. Sci. USA.

[bib57] Tsai A., Petrov A., Marshall R.A., Korlach J., Uemura S., Puglisi J.D. (2012). Heterogeneous pathways and timing of factor departure during translation initiation. Nature.

[bib58] Yamamoto H., Unbehaun A., Loerke J., Behrmann E., Collier M., Bürger J., Mielke T., Spahn C.M. (2014). Structure of the mammalian 80S initiation complex with initiation factor 5B on HCV-IRES RNA. Nat. Struct. Mol. Biol..

[bib59] Yusupova G.Z., Yusupov M.M., Cate J.H., Noller H.F. (2001). The path of messenger RNA through the ribosome. Cell.

[bib60] Zhou J., Lancaster L., Donohue J.P., Noller H.F. (2013). Crystal structures of EF-G-ribosome complexes trapped in intermediate states of translocation. Science.

