# Supplementary material for: Large-Scale Movements of IF3 and tRNA during Bacterial Translation Initiation
Source: Cell. 2016 Sep 22;167(1):133–144.e13. doi: 10.1016/j.cell.2016.08.074 (PMC5037330; doi:10.1016/j.cell.2016.08.074)
Supplement: Document S1. Tables S1–S3 [file mmc1.pdf]

Cell, Volume *167*

Supplemental Information

Large-Scale Movements of IF3 and tRNA during Bacterial Translation Initiation

Tanweer Hussain, Jose L. Llácer, Brian T. Wimberly, Jeffrey S. Kieft, and V. Ramakrishnan

Table S1. Refinement and model statistics, Related to Figure 1.

	PIC-1A	PIC-1B	PIC-1C	PIC-2A	PIC-2B	PIC-2C	PIC-3	PIC-4	PIC-III
**Model Composition**									
Non-hydrogen atoms	54057	54036	54058	55811	55695	55681	55727	55151	59482
Protein residues	4162	4160	4161	4251	4241	4234	4238	4166	4739
RNA bases	1534	1533	1534	1611	1608	1610	1611	1611	1611
**Refinement**									
Resolution used for refinement (Å)	3.6	4.4	5.4	4.3	4.5	5.2	4.25	4.15	5.1
Map sharpening B-factor (Å)	-87	-130	-157	-89	-101	-103	-107	-108	-180
Average B-factor (Å)	89.7	-	-	-	-	-	-	-	-
Fourier Shell Correlation (FSC)*	0.83	0.66	0.73	0.73	0.71	0.72	0.72	0.75	0.71
**Rms deviations**									
Bonds (Å)	0.006	0.008	0.008	0.007	0.065	0.008	0.007	0.006	0.006
Angles (°)	1.263	1.306	1.287	1.292	1.220	1.305	1.273	1.310	1.137
**Validation (proteins)**									
Molprobity score (Percentile in brackets)	3.36 (74th)	3.37 (75th)	3.33 (77th)	3.40 (74th)	3.25 (81st)	3.45 (71st)	3.43 (73rd)	3.53 (65th)	3.14 (84th)
Clashscore, all atoms (Percentile in brackets)	16.84 (97th)	16.05 (97th)	16.19 (97th)	17.23 (97th)	17.00 (97th)	18.85 (97th)	16.97 (97th)	19.27 (97th)	13.17 (97th)
Good rotamers (%)	60.67	61.19	62.28	60.24	64.93	60.45	59.12	55.20	66.80
**Ramachandran plot**									
Favored (%)	85.37	84.71	85.02	85.29	88.06	83.32	84.00	83.23	87.29
Outliers (%)	4.18	3.99	3.10	3.97	3.13	3.34	3.84	4.26	2.80
**Validation (RNA)**									
Correct sugar puckers(%)	96.48	96.02	96.41	96.15	96.64	96.28	96.65	96.46	95.16
Good backbone conformations(%)	70.27	68.75	70.34	71.64	71.71	69.96	72.26	69.09	72.33

* FSC= $\Sigma(N_{shell} FSC_{shell}) / \Sigma(N_{shell})$, where FSC_{shell} is the FSC in a given shell, N_{shell} is the number of "structure factors" in the shell. $FSC_{shell} = \Sigma(F_{model} F_{EM}) / (\sqrt{\Sigma(|F|^2_{model})} \sqrt{\Sigma(|F|^2_{EM})})$

Table S2. Local resolution of ligands, Related to Figure 1.

**Structures**	PIC-1A (Å)	PIC-1B (Å)	PIC-1C (Å)	PIC-2A (Å)	PIC-2B (Å)	PIC-2C (Å)	PIC-3 (Å)	PIC-4 (Å)	PIC-III (Å)
**Overall Resolution**	3.55	4.30	5.35	4.20	4.45	5.10	4.15	4.00	4.90
**IF3: NTD-linker**	4.55	6.10	7.00	5.10	6.20	8.30	6.70	6.95	8.50
**IF3: CTD**	3.90	5.20	6.00	4.90	6.20	6.95	5.10	5.25	5.80
**IF1**	4.05	5.20	6.45	4.65	6.20	6.55	4.85	-	5.45
**IF2**	-	-	-	-	-	-	-	-	6.95
**tRNA**	-	-	-	5.10	5.90	6.55	4.35	4.20	5.05
**ASL + mRNA(-4 to +4)**	-	-	-	4.85	4.60	5.45	4.10	3.95	4.95

Table S3. 30S head position in PICs, Related to Figure 2.

PICs	Degree of rotation of 30S head (degree of rotation)	Open/closed conformation of 30S
**PIC-1A**	Canonical position (0°)	Closed
**PIC-1B**	Swiveled position (~ 5°)	Closed
**PIC-1C**	(0°)	Open (30S head moves up)
**PIC-2A**	(~ 3 °)	Open (30S head moves up)
**PIC-2B**	Swiveled position (~ 9°)	Closed
**PIC-2C**	Canonical position (0°)	Closed
**PIC-3**	Canonical position (0°)	Closed
**PIC-4**	Canonical position (0°)	Closed
**PIC-I**	Canonical position (0°)	Closed
**PIC-II**	(~ 4°)	Closed
**PIC-III**	Canonical position (0°)	Closed
