# Supplementary figures and images for: Large-Scale Movements of IF3 and tRNA during Bacterial Translation Initiation
Source: Cell. 2016 Sep 22;167(1):133–144.e13. doi: 10.1016/j.cell.2016.08.074 (PMC5037330; doi:10.1016/j.cell.2016.08.074)

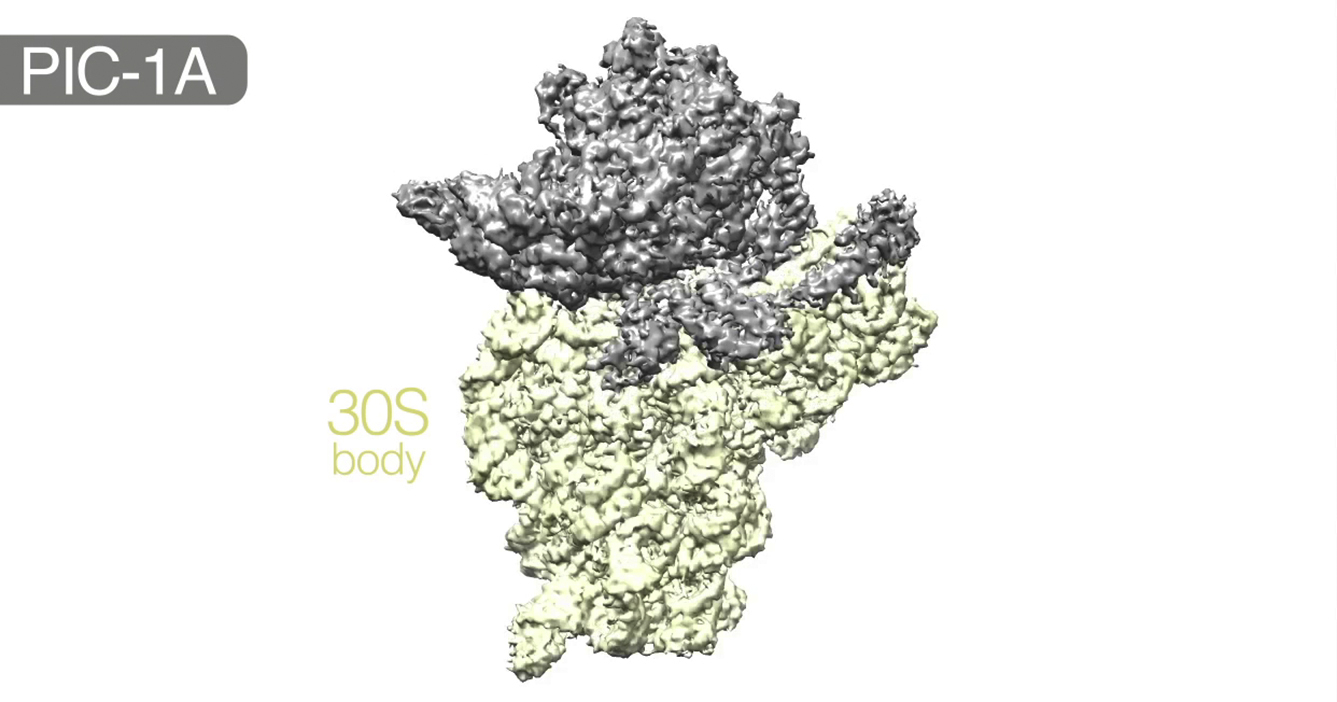

Supplement: Movie S1. Movie Showing Density for Ligands in the Various EM Maps: PICs 1A, 2A, 4, and III, Related to Figure 1 — Densities for all ligands are shown. In PIC-1A, a zoom to the P site is shown along with the density of mRNA and surrounding residues. In PIC-4 we also zoom into the codon:anticodon density. In PIC-III we show a zoomed view of C2 of IF2. [file mmc2.jpg]

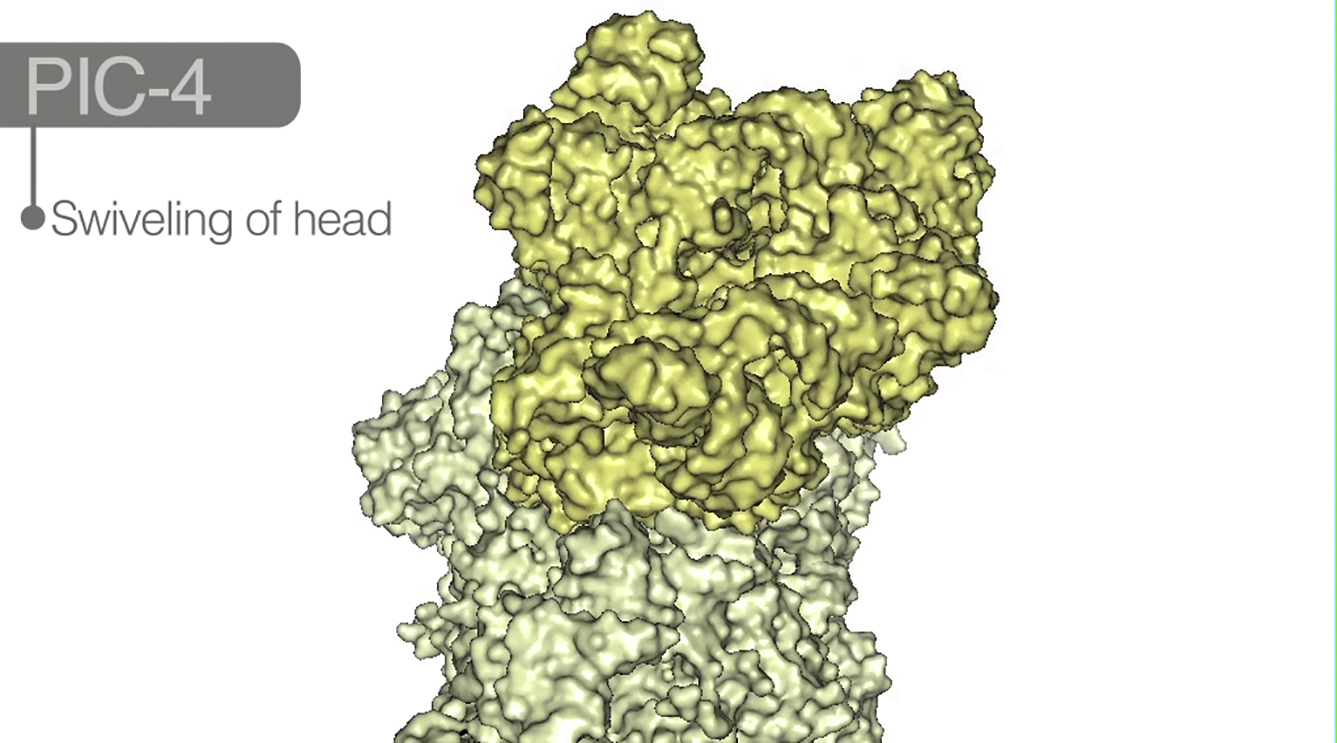

Supplement: Movie S2. Morphing of PICs 1C, 2B, and 4 to Highlight the Conformational Changes in the 30S Head with Respect to the Body, Related to Figure 2 — Both head swivel as well as upward movement of head (which opens up the mRNA latch) is shown. An mRNA (magenta) has been modeled in the mRNA channel. A zoomed view of opening (in PIC-1C) and closing of latch (PIC-2B and 4) is shown. In the neck of the 30S, h28, which relaxes (in PIC-1C) and compresses (PIC-4) during the 30S head movement is also shown. [file mmc3.jpg]

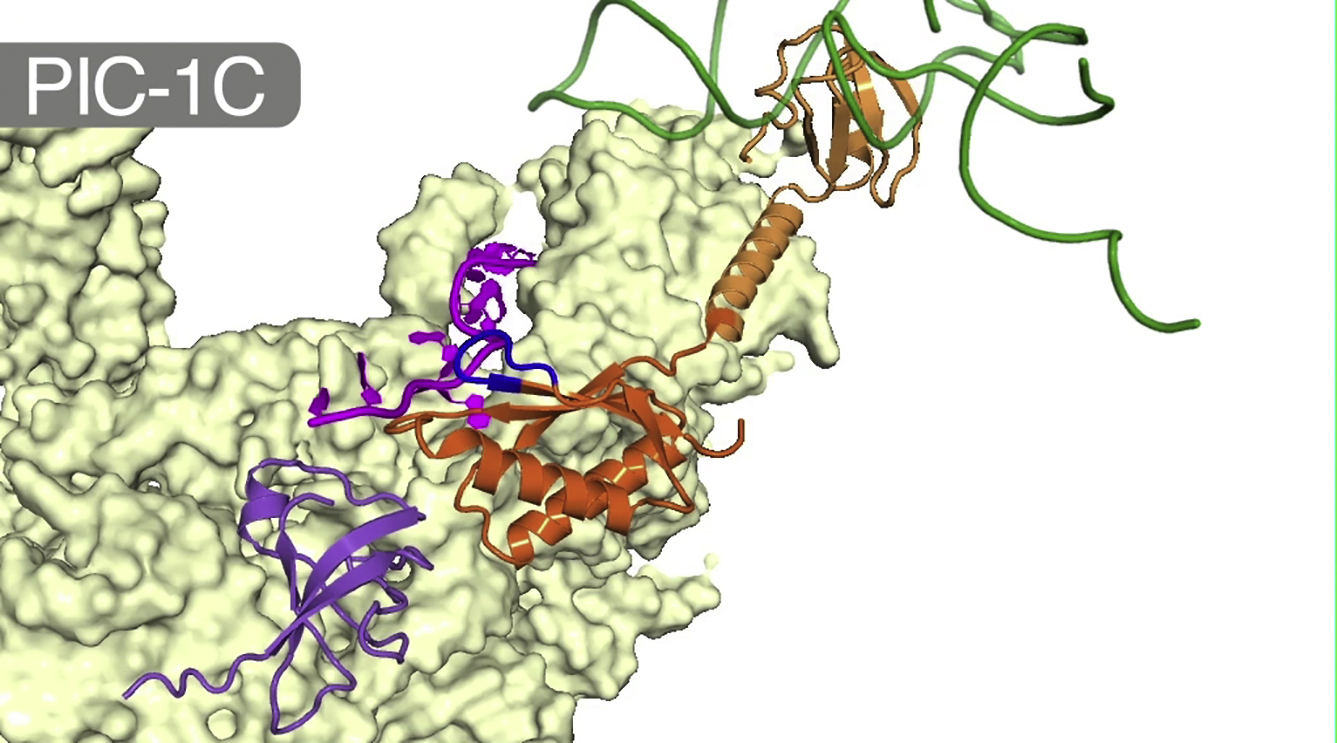

Supplement: Movie S3. Movie Highlighting the Conformational Changes in IF3 along the Initiation Pathway, Related to Figures 3 and 4 — The 30S head is not shown for the sake of clarity. A zoom to the P site shows the close positioning of IF3 to the mRNA in the absence of tRNA. The β-hairpin closer to tRNA is colored blue to highlight its movement. Surface representations of IF3 and tRNA in a few of the frames show the close positioning of these two ligands in certain steps. [file mmc4.jpg]

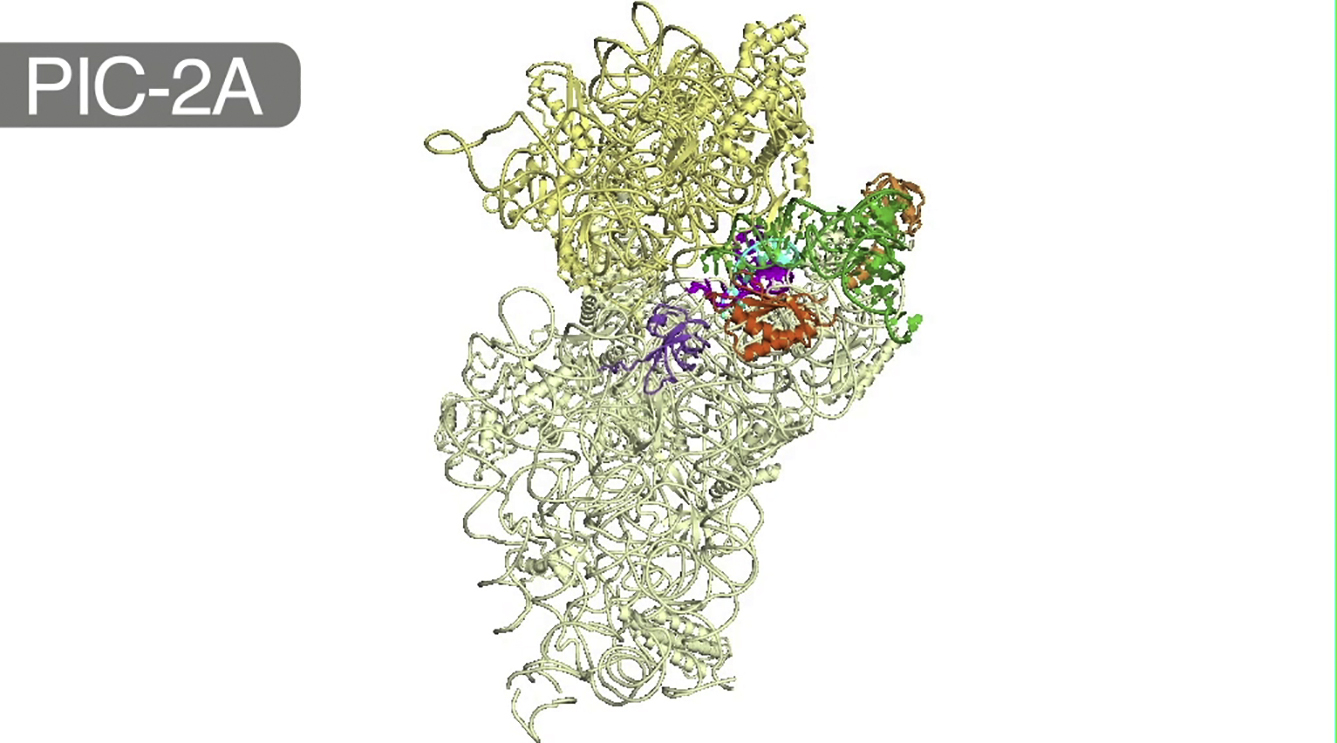

Supplement: Movie S4. Morphing of PICs 2A, 2B, and 2C to Highlight the Head Swivel, Leading to Movement of tRNA/mRNA toward the E Site, Related to Figure 5 — In the zoomed view a canonical E-site tRNA is shown in gray. During the transition from PIC-2A to 2B and subsequently to 2C, the codon in PIC-2A is shown in gray to highlight the movement of codon in PICs-2B and 2C. [file mmc5.jpg]

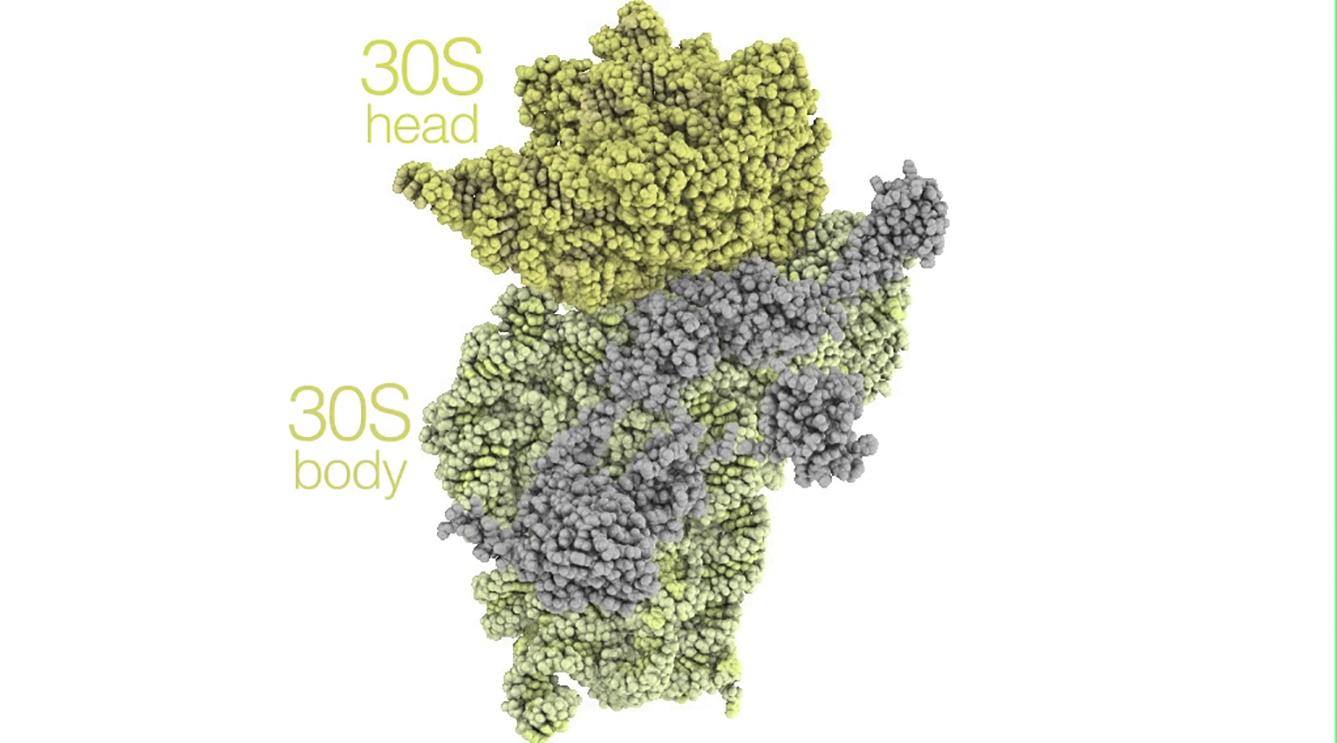

Supplement: Movie S5. Movie Showing the Complete Initiation Pathways, Related to Figures 6 and 7 — The movie starts with PIC-I. Thereafter we show the morph of PICs in this order: PIC- 1A, 1B, 1C, 2A, 2B, 2C, 3 and 4. In the end we show PIC-III and how C2 moves when 50S binds taking clue from recent 70S IC structure (Sprink et al., 2016). [file mmc6.jpg]
